# *TYK2* Promoter Variant and Diabetes Mellitus in the Japanese

**DOI:** 10.1016/j.ebiom.2015.05.004

**Published:** 2015-05-09

**Authors:** Seiho Nagafuchi, Yumi Kamada-Hibio, Kanako Hirakawa, Nobutaka Tsutsu, Masae Minami, Akira Okada, Katsuya Kai, Miho Teshima, Arisa Moroishi, Yoshikazu Murakami, Yoshikazu Umeno, Yasushi Yokogawa, Kazuhiko Kogawa, Kenichi Izumi, Keizo Anzai, Ryuichi Iwakiri, Kazuyuki Hamaguchi, Nobuhiro Sasaki, Sakae Nohara, Eiko Yoshida, Mine Harada, Koichi Akashi, Toshihiko Yanase, Junko Ono, Toshimitsu Okeda, Ryoji Fujimoto, Kenji Ihara, Toshiro Hara, Yohei Kikuchi, Masanori Iwase, Takanari Kitazono, Fumiko Kojima, Suminori Kono, Hironori Kurisaki, Shiori Kondo, Hitoshi Katsuta

**Affiliations:** a Department of Medical Science and Technology, Graduate School of Medical Sciences, Kyushu University, Fukuoka 812-8582, Japan; b Department of Medicine and Biosystemic Science, Graduate School of Medical Sciences, Kyushu University, Fukuoka 812-8582, Japan; c Department of Medicine and Clinical Sciences, Graduate School of Medical Sciences, Kyushu University, Fukuoka 812-8582, Japan; d Department of Pediatrics, Graduate School of Medical Sciences, Kyushu University, Fukuoka 812-8582, Japan; e Department of Diabetes and Metabolism, Fukuoka Red Cross Hospital, Fukuoka 815-8555, Japan; f Minami Masae Naika Clinic, Fukuoka 815-0071, Japan; g Okada Naika Clinic, Fukuoka 812-0053, Japan; h Department of Internal Medicine, Sawara Hospital, Sawara, Fukuoka 819-0002, Japan; i Yamaguchi Red Cross Hospital, Yamaguchi 753-8519, Japan; j Department of Diabetes, Oita Red Cross Hospital, Oita 870-0033, Japan; k Department of Internal Medicine, Hamanomachi Hospital, Fukuoka 810-8539, Japan; l Departmtent of Hepatology, Diabetes and Endocrinology, Saga University, Saga 849-8501, Japan; m Department of Internal Medicine & Gastrointestinal Endoscopy, School of Medicine, Saga University, Saga 849-8501, Japan; n Department of Medicine, School of Medicine, Oita University, 849-8501, Oita 879-5593, Japan; o Department of Clinical Laboratory Medicine, School of Medicine, Fukuoka University, Fukuoka 814-0180, Japan; p Department of Diabetology and Metabolism, School of Medicine, Fukuoka University, Fukuoka 814-0180, Japan; q Department of Internal Medicine, Shinkokura Hospital, Kitakyushu 803-8505, Japan; r National Institute of Health and Nutrition, Tokyo 162-8636, Japan; s Matsuyama Red Cross Hospital, Matsuyama 790-8524, Japan

**Keywords:** *Tyrosine kinase 2* (*TYK2*), Promoter variant, Diabetes mellitus, Virus, Polymorphism

## Abstract

**Background:**

Recently, natural mutation of *Tyrosine kinase 2* (*Tyk2*) gene has been shown to determine susceptibility to murine virus-induced diabetes. In addition, a previous human genome-wide study suggested the type 1 diabetes (T1D) susceptibility region to be 19p13, where the human *TYK2* gene is located (19p13.2).

**Methods:**

Polymorphisms of *TYK2* gene at the promoter region and exons were studied among 331 healthy controls, and 302 patients with T1D and 314 with type 2 diabetes (T2D) in the Japanese.

**Findings:**

A *TYK2* promoter haplotype with multiple genetic polymorphisms, which are in complete linkage disequilibrium, named *TYK2* promoter variant, presenting decreased promoter activity, is associated with an increased risk of not only T1D (odds ratio (OR), 2.4; 95% confidence interval (CI), 1.2 to 4.6; *P* = 0.01), but also T2D (OR, 2.1; 95% CI, 1.1 to 4.1; *P* = 0.03). The risk is high in patients with T1D associated with flu-like syndrome at diabetes onset and also those without anti-glutamic acid decarboxylase autoantibody.

**Interpretation:**

The *TYK2* promoter variant is associated with an overall risk for diabetes, serving a good candidate as a virus-induced diabetes susceptibility gene in humans.

**Funding:**

Ministry of Education, Culture, Sports, Science and Technology and of Health, Labor and Welfare of Japan.

## Highlights

• *TYK2* promoter variant with multiple polymorphisms was identified in the Japanese.
• *TYK2* promoter variant is associated with an increased risk of both type 1 and type 2 diabetes.
• *TYK2* promoter variant serves a good candidate as a virus-induced diabetes susceptibility gene in humans.

## 1 Introduction

Diabetes mellitus is on the rise worldwide, and is associated with improvement in socioeconomic conditions, increasing wealth, higher caloric and fat intake and lower physical activity (Scully, 2012, McCarthy, 2010). Accumulating evidence has also suggested the association of environmental factors such as toxins and viruses with diabetes (Jonietz, 2012). However, the role of these environmental factors in the development of diabetes is not yet fully understood.

Virus infection has long been considered to be a possible cause of type 1 diabetes (T1D), as suggested by many clinical and experimental observations (Taylor, 2013, Coppieters et al., 2012). It was reported that several viruses including coxsackie B virus, cytomegalovirus, varicella-zoster virus, and rubella virus, were found in the pancreatic islets in patients with severe fatal viral infections (Jenson et al., 1980), suggesting that systemic severe viral infections could lead to the pancreatic β-cell damage. Virus-induced diabetes is a more complex disease than previously thought, and is ascribed to diverse mechanisms that may lead to damage of the pancreatic β-cells (Coppieters et al., 2012). These mechanisms include direct virolysis, local inflammatory response, and triggering of autoimmunity against β-cells (Coppieters et al., 2012). However, the precise mechanisms of pancreatic β-cell damage caused by viral infections remain to be determined, and host factors that control virus-induced diabetes have not been elucidated.

Accumulating evidence strongly suggests the contribution of enteroviruses, which belong to the picornavirus group, to the elevated risk of diabetes (Tauriainen et al., 2011, Oikarinen et al., 2011, Tanaka et al., 2009). Since resistance to picornavirus infection has been shown to be dependent on innate immunity (Ida-Hosonuma et al., 2005, Takeuchi and Akira, 2009), the molecules regulating innate immune responses are candidates for determining susceptibility to virus-induced diabetes (Kounoue et al., 2008, Nagafuchi et al., 2013). These include interferon itself, interferon production and interferon receptor-mediated signaling pathway-associated molecules including pattern recognition receptors (PRR) directed against pathogen-associated molecular patterns (PAMPs) such as toll-like receptors (TLR) and intracellular helicases such as retinoic acid-inducible gene I (*RIG-I)* and interferon induced with the helicase C domain I (*IFIH1*) or melanocyte differentiation antigen (*MDA*)-*5* (*MDA-5/IFIH1*) (Takeuchi and Akira, 2009). Interferon-regulatory factors and interferon receptor-associated downstream molecules including *JAK1*, *TYK2*, *STAT1* and *STAT2* are also important with respect to serving as resistance against viral infections (Nagafuchi et al., 2013) (Supplementary Fig. 1). However, the exact host factors that confer susceptibility to virus-induced diabetes remain uncertain.

Since innate immunity plays a significant role in the protection against experimental encephalomyocarditis (EMC) virus (a picornavirus)-induced diabetes (Kounoue et al., 2008), it is suggested that intact operation of the interferon signaling pathway may be important for resistance against virus-induced diabetes. It should be noted that this experimental virus-induced diabetes in mice is an excellent model as rapid onset T1D including fulminant type, but not autoimmune diabetes (Imagawa et al., 2000, Nagafuchi et al., 2013). A separate study from our group presented experimental evidence that the *Tyk2* gene, an interferon receptor signaling pathway molecule, was responsible for encephalomyocarditis (EMC) virus-induced diabetes susceptibility in mice (Izumi et al., 2015). Highly virus-induced diabetes-susceptible strains such as SJL and SWR mice possessing a mutated *Tyk2* gene, which is associated with reduced expression of *Tyk2* gene in pancreatic β-cells, were prone to the development of diabetes caused by the diabetogenic strain of EMC-D virus (Izumi et al., 2015). Interestingly, a human genome-wide study suggested the T1D susceptibility region to be chromosome 19p13 (Mein et al., 1998), where the *TYK2* gene was located (19p13.2) (Firmbach-Kraft et al., 1990). However, the exact responsible gene has not yet been identified.

These observations suggest that the human *TYK2* gene may be associated with the risk for T1D and also confer a possible link with virus-induced diabetes susceptibility in humans. We thus examined the association of *TYK2* gene polymorphisms with T1D and type 2 diabetes (T2D), focusing on association with flu-like syndrome at diabetes onset.

## 2 Methods

### 2.1 Subjects

We studied 947 Japanese patients and controls. Those include 302 patients with T1D, 314 patients with T2D and 331 healthy controls. Among the 302 patients with T1D, 73 patients were associated with flu-like syndrome at the onset. Clinical profiles of the Japanese patients with T1D or T2D, and the healthy controls are presented in Table 1.

Patients were designated as T1D if fasting C-peptide was < 0.5 ng/ml and they were in an insulin-dependent condition (IDDM), or as T2D if fasting blood glucose levels were higher than 126 mg/dl and HbA1c levels exceeded 6.5% with non-insulin-dependent status (NIDDM). Patients with T1D were also grouped according to their age at onset, as 0 to 19 (0–19), 20 to 39 (20–39), 40 to 59 (50–59), and 60 to 79 (60–79) years old. The study was conducted according to the guidelines for human study and was approved by the ethical committee of the Kyushu University, Graduate School of Medical Sciences (No. 433-00). Written informed consent was obtained from all subjects including T1D, T2D and healthy controls involved in this study.

### 2.2 Genotyping of *TYK2* Gene

Genotyping had been performed to detect 25 exons and the putative promoter region, 1.3 kb upstream of start codon, of the *TYK2* gene. *TYK2* sequence reference was NCBI Reference Sequence: NG_007872.1. PCR amplification of the target genes, followed by the direct sequencing of the amplified gene, was conducted. A list of primers used to detect the polymorphisms of the *TYK2* gene is presented in Supplementary Table 1.

### 2.3 *TYK2* Promoter Variant Gene Analysis

To identify the *TYK2* promoter variant, we used PCR analysis followed by direct sequencing to identify − 930G > A and − 929 T > A at the promoter region, using the following primer sets: F:5′-GAA TCG CTT GAA TCC GGG AG-3′, and R:5′-ACC CTT CTT CTG TGC CAC AC-3′. Thus, we present *TYK2* promoter genotypes of wild type and variant type as GT and AA, respectively.

### 2.4 Statistical Analysis

The genotype distribution between the cases and controls was statistically assessed by *χ*^2^ test. Odds ratio (OR) and 95% confidence interval (CI) were estimated by Woolf's method. Statistical analysis was done using Stata version 10 (Stata Corporation, College Station, Texas).

## 3 Results

### 3.1 Polymorphisms of Human *TYK2* Gene

**Fig. 1:**
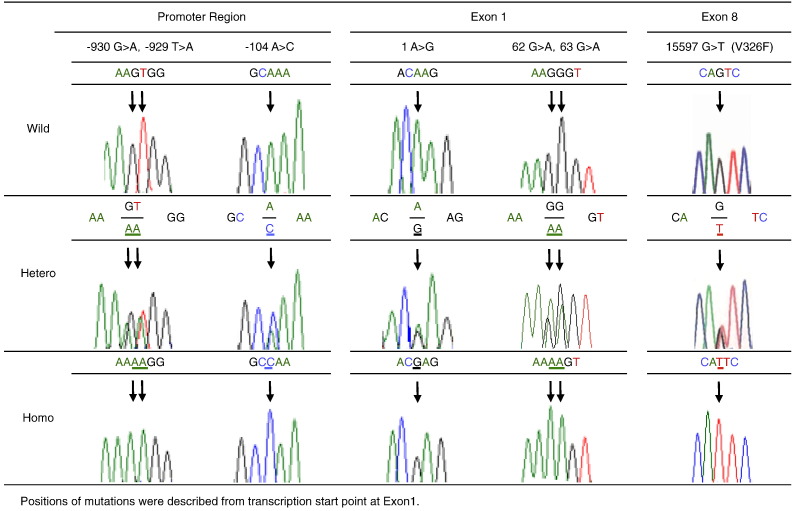
Polymorphisms of *TYK2* gene 22 patients with type 1 diabetes associated with flu-like syndrome at the onset. Positions of mutations were described from transcription start point at exon 1.

We first screened human *TYK2* gene polymorphisms in 22 patients with T1D associated with flu-like syndrome, suggestive of possible viral infection, by PCR amplification followed by direct sequencing. We found seven polymorphisms: − 930G > A, − 929T > A and − 104A > C at the promoter region from transcription start point at exon 1; 1A > G, 62G > A and 63G > A at exon 1, which is an untranslated region; and 15597G/T at exon 8 with an amino acid substitution from valine to phenylalanine (V326F) (Fig. 1).

Among them, 1A > G (rs17000728), 62G > A (rs17000728) and 63G > A (rs2304258) at exon 1, and 15597G/T (V326F) at exon 8 (rs2304256) have been already identified by the 1000 Genomes Project that included the Japanese population (The 1000 Genomes Project Consortium, 2010, The 1000 Genomes Project Consortium, 2012). Because the polymorphisms at the promoter region and exon 1 were in complete linkage disequilibrium in all 7 patients (Table 2), the haplotype was named *TYK2* promoter variant.

### 3.2 Significance of *TYK2* Promoter Variant

We first studied the association of the missense change at exon 8 with diabetes. We compared 244 patients with T1D, 255 patients with T2D and 254 healthy controls, and found no measurable difference in the genotype frequency between diabetic patients and healthy controls (Supplementary Table 2).

We further compared the prevalence of *TYK2* promoter variant; GT/AA and AA combined, and compared with wild type GT in 302 patients with T1D, 314 patients with T2D and 331 healthy controls (Table 1). Among them, 73 T1D patients had a flu-like syndrome at diabetes onset. The frequency of the *TYK2* promoter variant was significantly higher in patients with T1D (odds ratio (OR), 2.4; 95% confidence interval (CI), 1.2 to 4.6; *P* = 0.01), and also in patients with T2D (OR, 2.1; 95% CI, 1.1 to 4.1; *P* = 0.03), compared with healthy controls (Table 3). Thus, the *TYK2* promoter variant was more frequent in all patients with diabetes compared with healthy controls (OR, 2.3; 95% CI, 1.2 to 4.1; *P* = 0.009) (Table 3). The *TYK2* promoter variant was associated with a more evident increase in risk of T1D patients associated with a flu-like syndrome (OR, 3.6; 95% CI, 1.5 to 8.5; *P* = 0.005) (Table 3). In addition, the *TYK2* promoter variant was significantly more frequent among T1D patients without anti-glutamic acid decarboxylase autoantibody (GAD) (OR, 3.3; 95% CI, 1.6 to 7.2; *P* = 0.002), but not among anti-GAD autoantibody-positive patients (OR, 1.7; 95% CI, 0.8 to 3.9; *P* = 0.21) (Table 3).

It is thus suggested that the risk for diabetes conferred by the *TYK2* promoter variant is distinct from autoimmunity against pancreatic β-cells. We grouped TID associated with flu-like syndrome and analyzed the age at onset and anti-GAD antibody positivity, in association with *TYK2* promoter variant. There was no statistical significance in the age at onset (*P* = 0.16), but has significantly increased frequency in anti-GAD antibody negative T1D (OR, 5.0; 95% CI, 1.9 to 13.2; *P* = 0.0005) (Supplementary Table 3), consistent with the observation of all T1D patients. There was no gender difference in the frequency of *TYK2* promoter variant, among patients with T1D (male, 13/113; 11.5%, female, 16/189; 8.5%; *P* = 0.39), and also T2D (male, 14/161; 8.7%, female, 13/153; 8.5%; *P* = 0.95). In the age-specific analysis on subjects with T1D, we found that the *TYK2* promoter variant haplotype was associated with a higher risk for diabetes in younger people aged 0 to 19 years (OR, 2.4; 95% CI, 1.1 to 5.4; *P* = 0.04) and 20 to 39 years (OR, 3.1; 95% CI, 1.4 to 6.9; *P* = 0.006), but not in older patients aged 40–59 years (OR, 1.6; 95% CI, 0.5 to 5.1; *P* = 0.62) or 60–79 years (OR, 1.2; 95% CI, 0.1 to 9.5; *P* = 0.68) (Table 4).

Thus, the *TYK2* promoter variant is associated with an increased risk of T1D with a younger age of onset.

In the age group of 20–39, we found no difference in the age at onset (*P* = 0.17), however, those with variant type are more associated with anti-GAD negative people (OR, 5.1; 95% CI, 1.9 to 13.6; *P* = 0.0003), and also with flu-like syndrome at the onset (OR, 4.8; 95% CI, 1.4 to 15.49; *P* = 0.022) (Supplementary Table 3), consistent with the observation in all T1D. Since obesity is an important risk for endocrinological disorders (Dwivedi et al., 2012), we also analyzed the association between BMI and *TYK2* promoter variant in T2D. There was no difference in BMI between T2D with *TYK2* promoter wild type gene and variant type (*P* = 0.12) (Supplementary Table 4). In addition, there was increased *TYK2* promoter variant rate at statistical significance only in non-obese T2D with less than 26 BMI (OR, 2.4; 95% CI, 1.2 to 4.8; *P* = 0.01), but not obese T2D with more than 26 BMI (OR, 0.8; 95% CI, 0.2 to 3.7; *P* = 1.0) (Supplementary Table 4), suggesting that obesity is not likely involved in the increased risk associated with *TYK2* promoter variant in T2D.

### 3.3 Promoter Activity of the *TYK2* Promoter Variant

To determine the function of the *TYK2* promoter variant gene, we performed a luciferase assay, and found that the variant type promoter showed significantly reduced promoter activity (82.29 ± 0.03%; *P* < 0.001) (Supplementary Fig. 2A). Similarly, there was a slight decrease in the interferon-induced expressions of *TYK2* gene (mean ± SD of the relative expression; 0.59 ± 0.21) in peripheral blood mononuclear cells derived from diabetic patients with *TYK2* promoter variant (n = 14), compared with those (0.71 ± 0.28) of patients with the wild type *TYK2* gene (n = 17), while there was a mild increase in the interferon-induced expressions of *JAK1* gene (mean ± SD of the relative expression; before stimulation; 0.74 ± 0.19 to 0.82 ± 0.17 after stimulation) in peripheral blood mononuclear cells derived from diabetic patients with *TYK2* promoter variant (n = 14), compared with those (before stimulation; 0.82 ± 0.21 to 0.84 ± 0.21 after stimulation) of patients with the wild type *TYK2* gene (n = 17) (Supplementary Fig. 2B). The expression levels of ISGs, including *PKR*, *OAS* and *MxA*, induced by interferon stimulation in patients with *TYK2* promoter variant were also lower than those of patients with wild type ISGs (Supplementary Fig. 2C), which did not reach statistical significance (all: *P* > 0.05). These results suggest that the increased risk of developing diabetes conferred by the *TYK2* promoter variant may be due to reduced *TYK2* promoter activity accompanied by the decreased expression of the *TYK2* gene and ISGs, while increased expression level of *JAK1* gene on IFN stimulation in patients with TYK2 promoter variant may play a complementary role for the deteriorated *TYK2* gene expression to maintain ISGs responses. Further investigation is required to clarify the influence of *TYK2* promoter variant on cytokine responses in humans.

## 4 Discussion

In the present study, based on our experimental evidence that the natural susceptibility gene to EMC virus-induced diabetes was *Tyk2* in mice (Izumi et al., 2015), we could extend those observations to humans, with a *TYK2* promoter variant which is associated with an overall increased risk for diabetes in Japanese subjects, particularly in patients with T1D associated with flu-like syndrome at onset. In addition, a genome wide study had identified the T1D susceptibility-associated region as 19p13 (Mein et al., 1998), where the *TYK2* gene is located (19p13.2)(Firmbach-Kraft et al., 1990). All these observations taken together indicate that the *TYK2* gene might be regarded as a good candidate for the virus-induced diabetes susceptibility gene in humans. Surprisingly, the *TYK2* gene promoter region variant was more frequent not only in subjects with T1D but also those with T2D. If *TYK2* promoter variant is actually associated with increased susceptibility to virus-induced diabetes, these results suggest that viral infection may be one of the risk factors for developing T2D, which is consistent with the concept that the accumulation of environmental insults will lead to clinical diabetes (Toniolo et al., 1980). Since *TYK2* gene is also associated with other several cytokine signals including IL-6, IL-10, IL-12, and IL-23 (Strobl et al., 2011, Casanova et al., 2012, O'Shea et al., 2013), suggesting that deteriorated cytokine responses that can modulate immune/inflammatory reactions, alone or in combination, due to *TYK2* promoter variant, may also play a role to serve an increased risk for diabetes. Accordingly, it has been indicated to have a close link between inborn errors or polymorphisms of *TYK2* gene and a wide spectrum of autoimmune diseases, inflammatory diseases, tumors, and obesity (Strobl et al., 2011, Casanova et al., 2012, O'Shea et al., 2013, Derecka et al., 2012). Therefore unknown factors associated with the *TYK2* promoter variant other than viral infection may also contribute to increase the risk for diabetes. At least, as indicated in our study, *TYK2* promoter variant in T2D was associated with non-obese patients but not with obesity.

Interestingly, *TYK2* promoter variant was associated with a significantly higher susceptibility to diabetes in anti-GAD antibody-negative patients (OR, 3.3; *P* = 0.002), of which observation is consistent with *Tyk2* gene mutation dependent murine virus-induced diabetes that simulates non-autoimmune rapid onset and fulminant T1D without autoantibody production (Imagawa et al., 2000, Nagafuchi et al., 2013, Izumi et al., 2015). It was reported that the *Tyk2* gene played an important role not only in the interferon signaling pathway but also in the Th1 type immune response-associated IL-12-dependent signaling pathway (Shimoda et al., 2000). It was also reported that autoimmunity to pancreatic β-cells, associated with the development of T1D, was mainly dependent on Th1 type immune response (Haskins and Cooke, 2011). These observations suggest that the human *TYK2* gene promoter variant may reduce the risk for the development of Th1 type-dependent autoimmune reactivity to the pancreatic β-cells. In addition, if *TYK2* promoter variant confer risk for the development of diabetes due to increased susceptibility to viral-infection, possible induction of autoimmunity against pancreatic β-cells triggered by viral infections, which is a well-documented hypothesis (Fairweather and Rose, 2002, Stene et al., 2010), was not a major pathogenic mechanism, in *TYK2* promoter variant-associated susceptibility to type 1 diabetes. Since these data have been obtained in the Japanese population, less prone to T1D than other ethnic groups, the possible role of *TYK2* promoter variant needs to be verified in different populations.

In human cases the situation is highly different from that in experimental animals where mice have been infected with a virus and it is possible to prove that infection is causing diabetes, however, accumulation of circumstantial evidence to identify the putative virus-induced susceptibility gene in humans is important. It was reported that polymorphisms of the *IFIH1* gene, which is an intracellular pathogen recognition receptor for picornavirus including enteroviruses, operating as an inducer of interferon production (Takeuchi and Akira, 2009), is associated with risk or resistance for the T1D, serving possible virus-induced susceptibility gene in humans (Smyth et al., 2006, Nejentsev et al., 2009). Since the outcome of virus-induced diabetes is influenced by many factors including viral diabetogenicity and host susceptibility, the discovery of other risk genes associated with virus-induced diabetes in addition to *IFIH1* and *TYK2* genes is both possible and feasible. Unfortunately, at present, there is no appropriate assay system to prove the diabetogenicity of the virus infective for humans, fulfilling ‘Koch’s postulate (Tabrah, 2011). Mouse models that are simulative of human virus-induced diabetes, with higher virus-induced diabetes susceptibility for use as an in vivo assay system to evaluate the diabetogenic potential of the possible viral agents that are infectious for humans. Mouse strains endowed with high susceptibility to picornavirus-induced diabetes may be used as in vivo models to evaluate the diabetogenicity of candidate human viruses.

## Author Contributions

S.Na. designed the study, interpreted the data and wrote the manuscript. Y.K-H., K.Hi., K.Ka., M.T., A.M., E.Y., H.Ku, and H.Ka. performed the genetic analyses. N.T., M.M., A.O., Y.M., Y.U., Y.Y., K.K., K.I., K.An., R.I., K.Ha., N.S., S.No., K.Ak., T.Y., J.O., T.O., R.F., K.I., T.H., M.H., Y.K., M.I., T.K., F.K., H.Ka., S.Na., and S.Kondo collected the human samples of the patients and healthy controls, and analyzed the clinical data of the patients. S.Kono performed the statistical analysis.

## Conflict of Interest

There is no conflict of interest regarding this research.

## Figures and Tables

**Table 1:** Characteristics of patients with T1D and T2D, and healthy controls.

Characteristics	Type 1 diabetes^a^	Type 2 diabetes	Healthy controls
Number	302	314	331
Age (years) (range)	40.7 ± 17.3 (7–83)	61.8 ± 11.8 (17–91)	43.4 ± 12.7 (18–71)
Men (%)	37.4	51.3	53.5
HbA1c (%)^b^	8.9 ± 2.3	7.9 ± 1.6	5.2 ± 0.7
BMI (kg/m^2^)	21.8 ± 3.1	23.2 ± 3.6	22.0 ± 3.3
Age at diabetes onset (range)	27.8 ± 17.9 (0–73)	NA^c^	NA
Anti-GAD Ab^d^ positive (%)	58.6	NT^e^	NT

Values are means ± standard deviation.

T1D, type 1 diabetes; T2D, type 2 diabetes.

a Patients with T1D were diagnosed as they have fasting serum c-peptide level below 0.5 ng/ml.

b HbA1c (%) was expressed as National Glycohemoglobin Standardization Program (NGSP) value.

c NA: not available.

d Anti-GAD ab: anti-glutamic acid decarboxylase antibody (≥ 1.5 U/ml).

e NT: not tested.

**Table 2:** Screening of *TYK2* gene polymorphism in 22 patients with T1D associated with flu-like syndrome at diabetes onset.

Case no.	Age at the onset	Sex	SNPs at promoter region			SNPs at exon 1			SNP at exon 8
− 930G > A	− 929G > A	− 104A > C	1A > G	62G > A	63G > A	15597G > T
1	49	M							
2	47	F	Hetero	Hetero	Hetero	Hetero	Hetero	Hetero	
3	61	F	Hetero	Hetero	Hetero	Hetero	Hetero	Hetero	
4	31	F							
5	36	F							Hetero
6	40	F							Hetero
7	59	F							Hetero
8	9	M	Homo	Homo	Homo	Homo	Homo	Homo	
9	10	M							
10	43	M	Hetero	Hetero	Hetero	Hetero	Hetero	Hetero	
11	52	M	Hetero	Hetero	Hetero	Hetero	Hetero	Hetero	Hetero
12	53	M							Hetero
13	34	F	Hetero	Hetero	Hetero	Hetero	Hetero	Hetero	
14	24	F							Hetero
15	62	F							Hetero
16	35	M	Hetero	Hetero	Hetero	Hetero	Hetero	Hetero	Hetero
17	25	M							
18	30	M							
19	48	F							
20	40	F							
21	24	M							Hetero
22	27	M							Homo

T1D, type 1 diabetes.

Hetero: heterozygous polymorphism.

Homo: homozygous polymorphism.

**Table 3:**
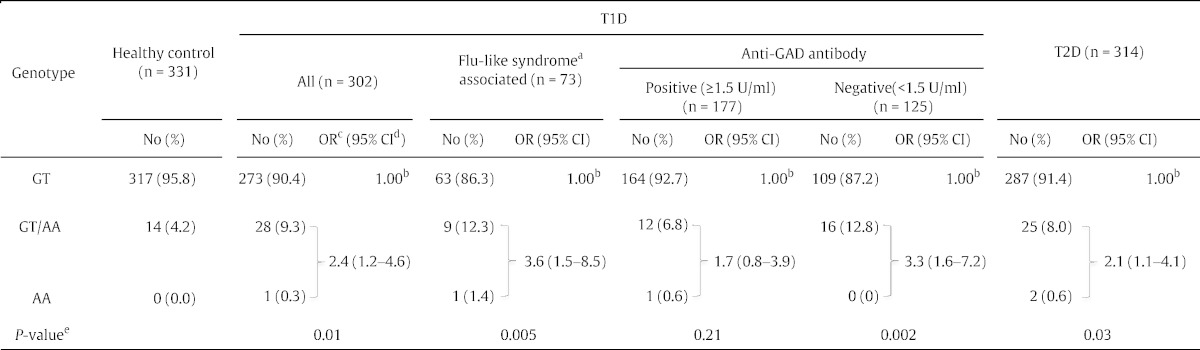
*TYK2* promoter variant in patients with T1D and T2D, and healthy controls.

T1D, type 1 diabetes; T2D, type 2 diabetes.

^a^ Symptoms of flu-like syndrome include fever, chills, sore throat, muscle and joint aches, poor appetite, diarrhea, cough, and fatigue, suggestive of certain viral infections.

^b^ Referent.

^c^ OR, odds ratio.

^d^ CI, confidence interval.

^e^ Heterozygous (GT/AA) and homozygous (AA) variant genotypes combined (*TYK2* promoter variant) versus homozygous wild genotype (GT) between the cases and healthy controls was statistically assessed by χ^2^ test.

**Table 4:** *TYK2* promoter variant genotypes in patients with T1D, with stratification by the age of onset.

Age at onset	T1D (n = 302)			OR^a^ (95% CI^b^)	*P*-value^c^
Wild	Hetero	Homo
0–19	104 (90.4%)	10 (8.7%)	1 (0.9%)	2.4 (1.1–5.4)	0.04
20–39	94 (87.9%)	13 (12.1%)	0 (0%)	3.1 (1.4–6.9)	0.006
40–59	56 (93.3%)	4 (6.7%)	0 (0%)	1.6 (0.5–5.1)	0.62
60–79	19 (95.0%)	1 (5.0%)	0 (0%)	1.2 (0.1–9.5)	0.68

T1D, type 1 diabetes.

a OR, odds ratio.

b CI, confidence interval.

c Heterozygous and homozygous variant genotypes combined (*TYK2* promoter variant) versus homozygous wild genotype in comparison with healthy controls (see Table 3).
