# Supplementary material for: TYK2 Promoter Variant and Diabetes Mellitus in the Japanese
Source: eBioMedicine. 2015 May 9;2(7):744–9. doi: 10.1016/j.ebiom.2015.05.004 (PMC4534683; doi:10.1016/j.ebiom.2015.05.004)
Supplement: Supplementary file 1 — Supplementary Material 1. [file mmc1.pdf]

Supplementary Information

1. Supplementary Methods Summary

1.1 Promoter Analysis of the *TYK2* Gene

The PCR-amplified *TYK2* promoter fragment, with either wild or variant type sequences, was cloned into a pGL4.17[luc2/Neo] vector (Promega). Insert was prepared from wild type *TYK2* or homozygous *TYK2* promoter variant by PCR amplification using forward primer 5'-AAAGCTAGCAGCTGCCCTGTGAGGAGGC-3' and reverse primer 5'-AAAAAGCTTTCCCCGCGGCTTCTTCCTGA-3', which led to a 1572bp product. Luciferase assay was conducted by transfection of vectors to 293T cells with 24-well plates. Luciferase activity was measured 24 hours after transfection in 293T cells using a dual luciferase assay kit (Promega Corporation, Madison, WI). The experiments were repeated five times.

1.2 Expression of *TYK2* gene and interferon stimulated genes (ISGs), including PKR, OSR and MxA gene

Patients with type 2 diabetes, possessing either *TYK2* wild type or promoter variant, were studied for the expression of *TYK2* gene, *JAK1* gene and interferon-stimulated genes before and after IFN-β stimulation. 14 patients with type 2 diabetes (age, 65.1±10.8; HbA1c, 7.3±0.8%) carrying heterozygous (n=12) and homozygous (n=2) *TYK2* promoter variant, and 17 patients with type 2 diabetes (age, 71.8±9.9; HbA1c, 7.1±0.6%) carrying wild type *TYK2* promoter were studied. The data are expressed as means±standard deviations.

PBMCs were isolated by LSM (MP BIOMEDICALS, Ohio, USA) from patients. PBMCs were stimulated with IFN-β (500U/ml) (SIGMA-ALDRICH, Missouri, USA) for 12h, after which total RNA was extracted using ISOGEN (Wako Chem., Tokyo). cDNA was synthesized from the RNA template (1 μg) with High-Capacity cDNA Reverse Transcription Kits (Applied Biosystems) according to the manufacturer protocol. Quantitative PCR was carried out by using an ABI 7500 real-time PCR system with Power SYBER green Master Mix (Applied Biosystems). The PCR was set up under the following thermal cycling conditions: 50℃ 2min, 95℃ 10min, followed by 40 cycles of 95℃ 15sec, and 63℃ 1 min. Fluorescence signals were collected by the machine using the extension phase of each PCR cycle. The threshold cycle value was normalized to that of β-actin. The qPCR was performed by using the following primer pairs: for human *TYK2* gene, 5'-TGGCATGAATCCTCGGGAAC-3' and 5'-CATGCTTGCCCTGCTCAAAG-3'; *JAK1* gene, 5'-CTACAGTCTGCACGGTTCGGA-3' and
5'-CGATCGAAACTCAGTTGGCTC-3'; Protein kinase R (PKR) gene, 5'-TCTGACTACCTGTCCTCTGGTTCT-3' and 5'-GCGAGTGTGCTGGTCACTAAAG-3'; 2'-5' oligoadenylate synthetase (OAS) gene, 5'-ACCTGGTTGTCTTCCTCAGTCC-3' and 5'-GAGCCTGGACCTCAAACTTCAC-3'; myxovirus resistance A (MxA) gene, 5'-TTCGGCTGTTTACCAGACTCC-3' and 5'-CAAAGCCTGGCAGCTCTCTAC-3'; β-actin gene, 5'-GCACCACACCTTCTACAATGAGC-3' and 5'-GGATAGCACAGCCTGGATAGCAAC-3'.

The experiments were repeated three times. The relative mRNA level was expressed as fold change relative to the value of the corresponding healthy non-diabetic control. Statistical analysis was done by Student's t-test.

2. Supplementary Figures

Supplementary Figure 1.

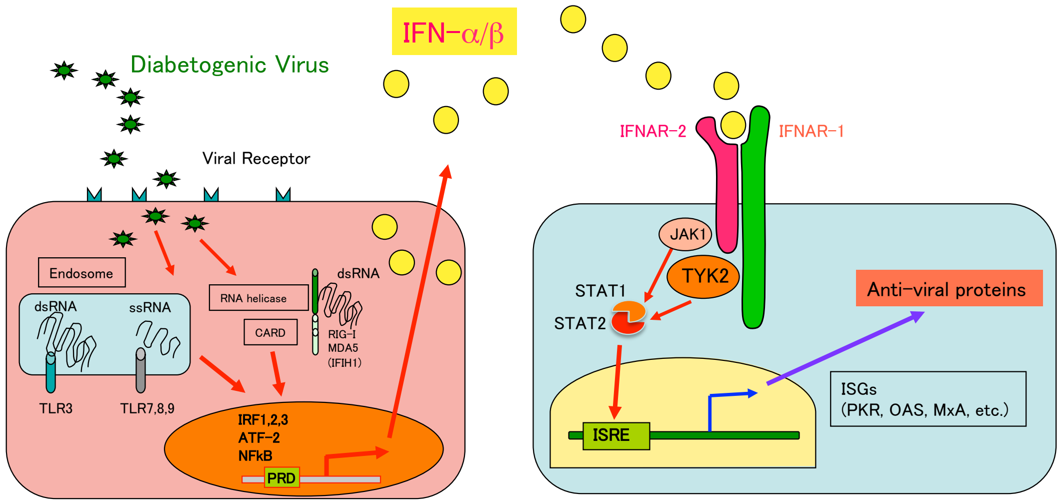

IRF: interferon regulatory factor
PRD: positive regulatory domain
CARD: caspase recruitment domain
TLR: Toll-like receptor
MDA: melanocyte differenciation antigen
(IFIH1: interferon induced with helicase C domain 1)
IFN: interferon

JAK1: Janus kinase 1
TYK2: Tyrosine kinase 2
STAT: Signal transducers and activator of transcription
IFNAR: IFN α/β receptor
ISRE: IFN stimulated response element
ISGs: IFN stimulated genes
PKR: RNA dependent protein kinase
OAS: 2',5'-oligoadenylate synthetase
MxA: IFN-induced GTP-binding protein MxA

Supplementary Figure 1. Type 1 interferon (IFN-α/β) production in response to putative diabetogenic virus infection and IFN signaling pathway.

JAK1 and TYK2 are reciprocal IFN receptor-associated molecules, mediating the downstream signal to induce IFN-stimulated genes (ISGs) to resist against viral infection. (modified from Diabetes and Viruses 2013, Springer Science-Media, p41, Fig.5.3)

Supplementary Fig. 2.

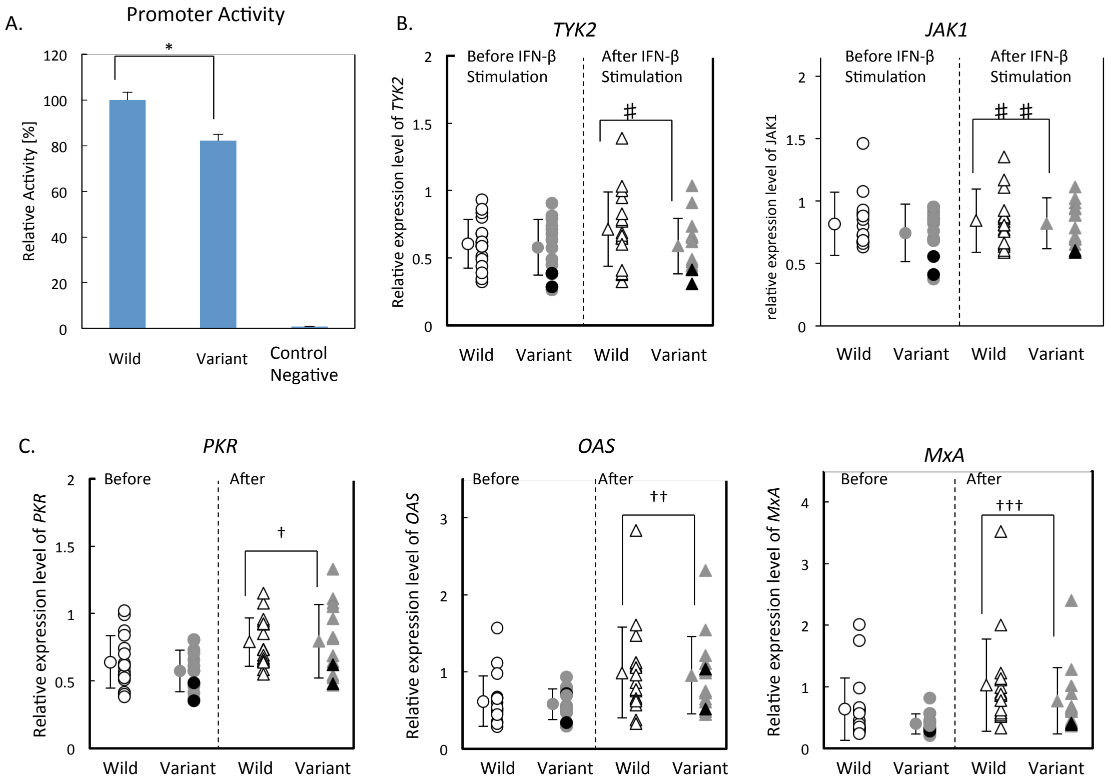

Supplementary Figure 2. Promoter assay of *TYK2* promoter variant and expression level of *TYK2*, *JAK1*, and interferon-stimulated genes.

Diabetic patients with heterozygous *TYK2* promoter variant type were compared with those with wild type *TYK2* gene. A. Promoter activity of *TYK2* promoter variant was assessed by the luciferase assay. Relative activity of the luciferase assay of *TYK2* promoter activity was expressed as percent, compared with that of wild type (100%). Mutated *TYK2* promoter variant showed significantly reduced promoter activity (82.6%±0.21) (n=9). (**P*<0.001) B. Relative expression level of *TYK2* gene induced by interferon-β (IFN-β) stimulation in patients with diabetes possessing heterozygous *TYK2* promoter variant (n=12) (●: before stimulation, ▲: after IFN-β stimulation) and homozygous patients (n=2) (●: before stimulation, ▲: after IFN-β stimulation) (before stimulation, 0.58±0.21; after stimulation, 0.59±0.21) compared with those with wild type gene (n=17) (○: before stimulation; 0.61±0.18, △: after IFN-β stimulation; 0.71±0.28). ($^{\#}P$=0.17). Relative expression level of *JAK1* gene induced by interferon-β (IFN-β) stimulation in patients with diabetes possessing heterozygous *TYK2* promoter variant (n=12) (●: before stimulation, ▲: after IFN-β stimulation) and homozygous patients (n=2) (●: before stimulation, ▲: after IFN-β stimulation) (before stimulation, 0.74±0.19; after stimulation, 0.82±0.17) compared with those with wild type gene (n=17) (○: before stimulation; 0.82±0.21, △: after IFN-β stimulation; 0.84±0.21) ($^{\#\#}P$=0.781). C. Relative expression level of ISGs induced by interferon-β (IFN-β) stimulation in patients with diabetes possessing heterozygous *TYK2* promoter variant (n=12) (●: before stimulation, ▲: after stimulation) and homozygous patients (n=2) (●: before stimulation, ▲: after stimulation) compared with those with wild type gene (n=17) (○: before stimulation, △: after stimulation). Results of relative expressions of ISGs are shown in patients with heterozygous and homozygous *TYK2* promoter variants. Before stimulation: *PKR*, 0.57±0.15; *OAS*, 0.58±0.20; *MxA*, 0.40±0.17. After stimulation: *PKR*, 0.79±0.27; *OAS*, 0.96±0.50; *MxA*, 0.77±0.54. In patients with wild type *TYK2* gene: before stimulation: *PKR*, 0.64±0.19; *OAS*, 0.62±0.33; *MxA*, 0.64±0.51. After stimulation: *PKR*, 0.79±0.18; *OAS*, 0.99±0.59; *MxA*, 1.02±0.75. The data are expressed as means ± standard deviations. ($^{\dagger}P$=0.94, $^{\dagger\dagger}P$=0.87, $^{\dagger\dagger\dagger}P$=0.30)

3. Supplementary Tables

Supplementary Table 1. Set of primers for amplification of *TYK2* gene.

	Forward	Reverse
Promoter Region	5'-GCCAGACCCCATCTCTACAAA-3'	5'-GGGAACACAAGCTCGAACC-3'
Exon 1	5'-AATCGCGGCTGAGTGACGAATG-3'	5'-GACCCAGACCCAGCTTTGAAGA-3'
Exon 2	5'-CTGGACATAAACTCTCCTAGGC-3'	5'-GACCATCTTGACCAACATGGTG-3'
Exon 3	5'-GTGGGTGGAAGGTTGAAGAG-3'	5'-GTGGATAGACGGATGGATGG-3'
Exon 4	5'-GGCTGACGGTAGCAAATGAC-3'	5'-CTGGGGCTTAGCACAGAGTC-3'
Exon 5	5'-GAAGCTGGTCTGACTCTGTGC-3'	5'-GCCCCTAAGTCTCCCACAA-3'
Exon 6	5'-CTCTGGGCTAGAGAGGAACG-3'	5'-GTCTACCCTGGCTCCCAGAT-3'
Exon 7	5'-ACCTGGCTAGTGTGCCTGTT-3'	5'-TCAGAGGCTAGGGTCAAGGA-3'
Exon 8	5'-GGAGGTATAAACGGGCATTG-3'	5'-GGAAATAGCCGTCCACCAG-3'
Exon 9	5'-GTAGGGGCTGGGCTAGGG-3'	5'-CCCCTAGGGCTCACAGTCTA-3'
Exon 10	5'-GGGTATGGGTCCAGAGTGG-3'	5'-GCAGAGGTGGGAGCAGTAAG-3'
Exon 11	5'-TACCGCCTGATCCTCACAGT-3'	5'-GCAGGCATCAAGTCATGGAG-3'
Exon 12	5'-GTGGGATGTGGCATCTCTCC-3'	5'-TGAAAGTTAGCAGCTGATCTCC-3'
Exon 13	5'-TGGGAGATCAGCTGCTAACTT-3'	5'-GCCACCTCCTCCACAGAC-3'
Exon 14	5'-GTGTGTCCGTGGAGGAGGT-3'	5'-GAGGGTTGGGGTACAGATCA-3'
Exon 15	5'-ATCCAGAGGGCAGAAGCAG-3'	5'-AGGCTGGTCTCGAACTCCTG-3'
Exon 16	5'-GTTGGCGTCTGTGCCTCT-3'	5'-GCGAAAGGAGCAGGGGAAG-3'
Exon 17	5'-CTTCCCCTGCTCCTTTCAC-3'	5'-AGAAGGGATGCAGCTTTGAG-3'
Exon 18	5'-GACTCCTCTGGGTCCCTTTC-3'	5'-CCTCTCGTGCGCTATAGGCA-3'
Exon 19	5'-TTTGTGACTCCCAAGTGTGG-3'	5'-CTCAACCCCCCAAACTCCTTC-3'
Exon 20	5'-CACCCACGCTCTAACCACGC-3'	5'-TGGTGCAGGGATTGGGGAGG-3'
Exon 21	5'-CTCTGCTGGGCTCAAGGTAG-3'	5'-CCCAAGCTGAAGAGGAAGG-3'
Exon 22	5'-CTCCTGGCTGCTCAGGTC-3'	5'-CTGGGATCATGCCCTATCAT-3'
Exon 23	5'-GATCCCCAAGCCCTCAGT-3'	5'-CCCAGCCTATGCCTTTCTAA-3'
Exon 24	5'-GCTGGGATTACAGGCATGAG-3'	5'-CCCTCTCCACAGCAGGATAG-3'
Exon 25	5'-CCTTTGTCTTTCCCTGACCC-3'	5'-CAGGGCTGCCATTGTGCCTC-3'

Supplementary Table 2. SNP at *TYK2* Exon 8 in patients with T1D, T2D and healthy controls.

SNP at Exon 8 (15597G/T)	Healthy Controls (n=254)	Type 1 DM				Type 2 DM (n=255)	
		All (n=244)		Flu-like syndrome[*] associated (n=36)			
	No (%)	No (%)	OR[#] (95% CI)	No (%)	OR[#](95% CI)	No (%)	OR[#](95% CI)
GG	115 (45.3%)	103 (42.2)	1.00[¶]	18 (50.0)	1.00[¶]	96 (37.6)	1.00[¶]
GT	116 (45.7%)	104 (42.6)	1.1 (0.8–1.6)	12(33.3)	0.8 (0.4–1.7)	121 (47.5)	1.3 (0.9–1.9)
TT	23 (9.0%)	37 (15.2)		6 (16.7)		38 (14.9)	
P value[†]		0.49		0.59		0.08	

[*]Symptoms of flu-like syndrome includes fever, chills, sore throat, muscle and joint aches, poor appetite, diarrhea, cough, and fatigue, suggestive of certain viral infections.

[¶]referent, [#]OR, odds ratio; [§]CI,confidence interval

[†]Heterozygous and homozygous variant genotypes combined versus homozygous wild genotype.

Supplementary Table 3. *TYK2* promoter variant in patients with T1D and with flu-like syndrome at the onset and of age from 20 to 39.

Genotype	TT1D (n=302)			T1D associated with flu-like syndrome (n=73)							T1D age 20-39 (n=107)						
				All#		age at onset (mea±SD)	Anti-GAD antibodyʃ				age at onset (mea±SD)	Anti-GAD antibodyʃ				with flu-ike syndrome (n=23)	
							Positive (≥1.5U/ml) (n=34)		Negative (<1.5U/ml) (n=39)			Positive (≥1.5U/ml) (n=69)		Negative (<1.5U/ml) (n=38)			
	No (%)	OR# (95% CI§)	age at onset (mea±SD)	No (%)	OR (95% CI)		No (%)	OR (95% CI)	No (%)	OR (95% CI)		No (%)	OR (95% CI)	No (%)	OR (95% CI)	No (%)	OR (95% CI)
GT	273 (90.4)	1.00¶	28.0±18.1	63 (86.3)	1.00¶	25.3±17.2	31(91.4)	1.00¶	32(82.1)	1.00¶	28.2±5.6	63(91.3)	1.00¶	31(81.6)	1.00¶	19(82.3)	1.00¶
GT/AA	28 (9.3)		26.4±15.8	9 (12.3)		36.9±12.7	2(5.9)		7(18.0)		30.5±6.3	6(8.7)		7(18.4)		4(17.4)	
		2.4 (1.2-4.6)	25.6±16.2		3.6 (1.5-8.5)	33.5±16.0		2.2(0.6-8.0)		5.0(1.9-13.2)	30.5±6.3		2.1(0.8-5.8)		5.1(1.9-13.6)		4.8(1.4-15.9)
AA	1 (0.3)		3.0.	1 (1.4)		3.0.	1(2.9)		0(0.0)		0(0.0)	0(0.0)		**0(0.0)**		0(0.0)	
P-value	0.01♮		0.48‡	0.005♮		0.16‡	0,20ʃ		P=0.0005ʃ		P=0.17‡	P=0.12ʃ		P=0.0003ʃ		0.022ʃ	

*Symptoms of flu-like syndrome include fever, chills, sore throat, muscle and joint aches, poor appetite, diarrhea, cough, and fatigue, suggestive of certain viral infections.

¶referent, #OR, odds ratio; §CI,confidence interval

ʃHeterozygous (GT/AA) and homozygous (AA) variant genotypes combined (TYK2 promoter variant) versus homozygous wild genotype (GT) between the cases and healthy controls was statistically assessed by χ 2 test. When the number of the patients of the group was less than 5, Fisher's exact test was used.

‡Statictical significance regarding age at onset between the wild and variant type were calculated by Student's t test.

♮See Table 3.

T1D, type 1 diabetes.

Supplemental Table 4. *TYK2* promoter variant and obesity in patients with T2D.

Genotype	T2D (n=314)						
	ALL		BMI* (kg/m2)				
			ALL	≤26(n=257)		>26(n=57)	
	No (%)	OR (95% CI)		No (%)	OR (95% CI)	No (%)	OR (95% CI)
GT	287 (91.4)	1.00¶	23.3±3.8	232(90.3)	1.00¶	55(96.5)	1.00¶
GT/AA	25 (8.0)			24(9.3)		2(3.5)	
		2.1 (1.1-4.1)	22.4±2.3		2.4(1.2-4.8)		0.8(0.2-3.7)
AA	2 (0.6)			1(0.4)		0(0.0)	
P-value	0.03ʃ		0.12‡	0.01ʃ		1.0ʃ	

*BMI: body mass index

¶referent, #OR, odds ratio; §CI,confidence interva

ʃHeterozygous (GT/AA) and homozygous (AA) variant genotypes combined (TYK2 promoter variant) versus homozygous wild genotype (GT) between the cases and healthy controls was statistically assessed by χ 2 test. When the number of the patients of the group was less than 5, Fisher's exact test was used.

‡Statictical significance regarding age at onset between the wild and variant type were calculated by Student's t test.
