# Supplementary material for: TYK2 Promoter Variant and Diabetes Mellitus in the Japanese
Source: eBioMedicine. 2015 May 9;2(7):744–9. doi: 10.1016/j.ebiom.2015.05.004 (PMC4534683; doi:10.1016/j.ebiom.2015.05.004)
Supplement: Supplementary file 2 — Supplementary Material 2. [file mmc2.pdf]

nature COMMUNICATIONS

ARTICLE

Received 28 Dec 2014 | Accepted 24 Feb 2015 | Published 7 Apr 2015

DOI: 10.1038/ncomms7748

OPEN

Reduced *Tyk2* gene expression in β-cells due to natural mutation determines susceptibility to virus-induced diabetes

Kenichi Izumi[1,2,3,*], Keiichiro Mine[1,*], Yoshitaka Inoue[1], Miho Teshima[1], Shuichiro Ogawa[1], Yuji Kai[1], Toshinobu Kurafuji[1], Kanako Hirakawa[1], Daiki Miyakawa[1], Haruka Ikeda[1], Akari Inada[4], Manami Hara[5], Hisakata Yamada[6], Koichi Akashi[2], Yoshiyuki Niho[2], Keisuke Ina[7], Takashi Kobayashi[8], Yasunobu Yoshikai[6], Keizo Anzai[3], Teruo Yamashita[9], Hiroko Minagawa[9], Shuji Fujimoto[1], Hironori Kurisaki[1], Kazuya Shimoda[10], Hitoshi Katsuta[1] & Seiho Nagafuchi[1]

Accumulating evidence suggests that viruses play an important role in the development of diabetes. Although the diabetogenic encephalomyocarditis strain D virus induces diabetes in restricted lines of inbred mice, the susceptibility genes to virus-induced diabetes have not been identified. We report here that novel *Tyrosine kinase 2 (Tyk2)* gene mutations are present in virus-induced diabetes-sensitive SJL and SWR mice. Mice carrying the mutant *Tyk2* gene on the virus-resistant C57BL/6 background are highly sensitive to virus-induced diabetes. *Tyk2* gene expression is strongly reduced in *Tyk2*-mutant mice, associated with low *Tyk2* promoter activity, and leads to decreased expression of interferon-inducible genes, resulting in significantly compromised antiviral response. *Tyk2*-mutant pancreatic β-cells are unresponsive even to high dose of Type I interferon. Reversal of virus-induced diabetes could be achieved by β-cell-specific *Tyk2* gene expression. Thus, reduced *Tyk2* gene expression in pancreatic β-cells due to natural mutation is responsible for susceptibility to virus-induced diabetes.

[1] Department of Medical Science and Technology, Graduate School of Medical Sciences, Kyushu University, Fukuoka 812-8582, Japan. [2] Department of Medicine and Biosystemic Science, Graduate School of Medical Sciences, Kyushu University, Fukuoka 812-8582, Japan. [3] Department of Hepatology, Diabetes and Endocrinology, School of Medicine, Saga University, Saga 849-8501, Japan. [4] Department of Diabetes and Genes, Graduate School of Medical Sciences, Kyushu University, Fukuoka 812-8582, Japan. [5] Department of Medicine, University of Chicago, Chicago, Illinois 60637, USA. [6] Division of Host Defense, Research Center for Prevention of Infectious Diseases, Medical Institute of Bioregulation, Kyushu University, Fukuoka 812-8582, Japan. [7] Department of Molecular Anatomy, Faculty of Medicine, Oita University, Oita 879-5593, Japan. [8] Department of Infectious Diseases Control, Faculty of Medicine, Oita University, Oita 879-5593, Japan. [9] Department of Microbiology and Medical Zoology, Aichi Prefectural Institute of Public Health, 7-6 Nagare, Tsujimachi, Kita-ku, Nagoya, Aichi 462-8576, Japan. [10] Division of Gastroenterology and Hematology, Department of Internal Medicine, Faculty of Medicine, University of Miyazaki, Miyazaki 889-1692, Japan. * These authors contributed equally to this work. Correspondence and requests for materials should be addressed to S.N. (email: nagafu_s@med.kyushu-u.ac.jp).

© 2015 Macmillan Publishers Limited. All rights reserved.

Diabetes mellitus is on the rise worldwide, and is associated with improvement in socioeconomic conditions, increasing wealth, higher caloric and fat intake and lower physical activity[1]. Accumulating evidence has also suggested the association of environmental factors such as toxins and viruses with diabetes[2,3]. From the middle of the last century, it has been recognized that a number of viruses are closely associated with the onset of diabetes in man and animals[4,5]. Recent human type 1 diabetes and viral infection research has suggested the closest link with enteroviruses[6–9], which belong to the picornavirus group. In experimental animals, diabetes was described to be induced by encephalomyocarditis (EMC) virus (also a member of the picornavirus group), cytomegalovirus, mengovirus and kilham rat virus[10]. Among these viruses, EMC virus-induced diabetes has provided an excellent animal model for studying virus-induced diabetes, and the pathogenesis of EMC virus-induced diabetes in mice has been extensively studied[10–12]. Development of diabetes induced by EMC virus has been shown to be influenced by the strain and challenge dose of the virus, and also host factors including sex, immunoprotective function, inflammatory responses with macrophages, cytokines, chemokines, chemical mediators and genetic background[10–12].

Yoon *et al.* successfully isolated the highly diabetogenic D variant of EMC (EMC-D) virus and the nondiabetogenic B strain of the EMC (EMC-B) virus by the plaque clone purification method[13]. Even diabetogenic EMC-D virus induces diabetes only in a limited number of mouse strains, such as DBA, SJL and SWR mice, while A/J and BALB/c mice are moderately susceptible[10,12,14–17]. C57BL/6, CBA, AKR and C3H/He mice are all resistant to EMC-D virus-induced diabetes[10,12,14–17]. It was reported that a single autosomal-recessive gene, which is inherited in a Mendelian manner, controls susceptibility to the virus[18]. However, the susceptibility gene(s) to EMC virus-induced diabetes in the highly sensitive mice has not been identified in spite of initial recognition of strain-specific susceptibility to EMC virus-induced diabetes more than 40 years ago[16,19].

It is thought that innate immunity, such as interferon (IFN), macrophages and early inflammatory responses, most likely determines outcomes after EMC-D virus infection, since EMC-D virus-induced diabetes develops within 4 days after infection, and infection to T-lymphocyte-deficient or B-lymphocyte-deficient mice did not make the mice more susceptible to virus-induced diabetes[15,17]. Recent advances in the field of innate immunity have elucidated the significance of pattern recognition receptors directed against pathogen-associated molecular patterns[20]. These include Toll-like receptors (TLR), intracellular helicase such as melanocyte differentiation-associated protein 5 (MDA5; or IFN induced with helicase C domain 1 (IFIH1)) for picornavirus, retinoic acid inducible gene I for paramyxovirus and intracellular DNA receptors[20–22]. McCartney *et al.*[23] reported that MDA5 and TLR3 are both required to activate IFN-dependent antiviral responses, in preventing EMC-D virus-induced diabetes in mice. In addition, IFN receptor (IFNR)-associated molecules including Tyrosine kinase 2 (Tyk2), Janus kinase 1 (Jak1), Signal Transducers and Activator of Transcription (Stat) 1 and Stat2 are all involved in IFNR-mediated downstream signalling, operating IFN-dependent antiviral responses (Fig. 1).

These observations taken together suggest that molecules involved in innate immunity could serve as candidate genes that determine the susceptibility of sensitive strains of mice to virus-induced diabetes. Interestingly, deficiency of the *Tyk2* gene results in a reduced antiviral response[24]. In addition, the human *TYK2* gene was mapped to the possible type 1 diabetes susceptibility locus[25].

We report here that novel *Tyk2* gene mutations are present in virus-induced diabetes-sensitive strains of mice, associated with significantly reduced *Tyk2* gene expression along with extensively deteriorated antiviral activity in β-cells, leading to the increased susceptibility to virus-induced diabetes. Thus, reduced *Tyk2* gene expression in pancreatic β-cells due to natural mutation is responsible for susceptibility to virus-induced diabetes.

Results

Tyk2 KO mice and virus-induced diabetes. We carried out intraperitoneal challenge with 1.0×10^3 plaque-forming unit of EMC-D virus to *Tyk2* gene knockout (*Tyk2* KO) and wild-type (*Tyk2* WT) male mice with virus-induced diabetes-resistant C57BL/6J (B6) background, and measured blood glucose levels. As shown in Fig. 2a, *Tyk2* KO mice intraperitoneally injected with EMC-D virus exhibited high blood glucose levels exceeding 14 mmol l^{-1} on the fourth day after infection and the high blood glucose levels persisted thereafter. As controls, we also used a nondiabetogenic strain of EMC-B virus, and found that the virus did not induce diabetes even in *Tyk2* KO mice. Intravenous injection of the virus induced diabetes as well as intraperitoneal challenge, while oral administration of the virus could not induce diabetes (Supplementary Fig. 1). Thus, it was concluded that *Tyk2* KO mice had high susceptibility to EMC-D virus-induced diabetes, challenged by intraperitoneal route. We also confirmed low circulating insulin and high glucose levels in *Tyk2* KO mice infected with EMC-D virus along with the reduced insulin content of the pancreas (Fig. 2b). It was concluded that insulin-producing capability of pancreatic β-cells of *Tyk2* KO mice infected with EMC-D virus were significantly decreased. The virus titres in the pancreas of *Tyk2* KO mice showed longer persistence and was at a significantly higher level from 1 to 5 days after infection in comparison with *Tyk2* WT mice, with ∼60-fold differences between the two groups on day 5 (Fig. 2c), suggesting that increased proliferation of the EMC-D virus in the pancreas of *Tyk2* KO mice might lead to the extensive damage of the pancreatic β-cells.

Importance of _Tyk2_ gene expression in β-cell. In order to clarify whether expression of *Tyk2* is important in immunocompetent cells or in parenchymal cells, we set up a splenic chimera mouse system. The irradiated *Tyk2* KO recipient mice that were transferred spleen cells derived from either WT donor mice or *Tyk2* KO mice developed diabetes 7 days after EMC-D virus infection, whereas the irradiated WT recipient mice received spleen cells derived from either WT donor mice or *Tyk2* KO mice did not develop diabetes (Fig. 2d). All irradiate diabetic *Tyk2* KO mice that received WT or KO spleen cells died by 14 days after infection, while WT mice receiving either WT or *Tyk2* KO mice derived spleen cells could escape from the development of diabetes (Fig. 2d), and could survive until 3 weeks after infection. Irradiated mice presented deteriorated general condition, and tended to succumb to EMC-D virus infection. Taken overall, these results indicate that *Tyk2* expression in parenchymal cells, not in splenic immune cells, is important to resist against EMC-D virus-induced diabetes.

To determine the significance of *Tyk2* gene expression in pancreatic β-cells among parenchymal cells, we generated transgenic (Tg) B6 mice that have mouse *insulin* promoter (*MIP*) driving *Tyk2* transgene vector (*MIP-Tyk2*)[26]. As shown in Fig. 2e, while *Tyk2* KO mice developed hyperglycaemia, *MIP-Tyk2* Tg *Tyk2* KO mice could maintain normal blood glucose levels. Consistent with this, pancreatic islets of *MIP-Tyk2* Tg *Tyk2* KO mice remained histologically intact with minimal destructive changes after viral infection compared with extensive damaged islets in *Tyk2* KO mice (Fig. 2f). These results suggested that *Tyk2*

© 2015 Macmillan Publishers Limited. All rights reserved.

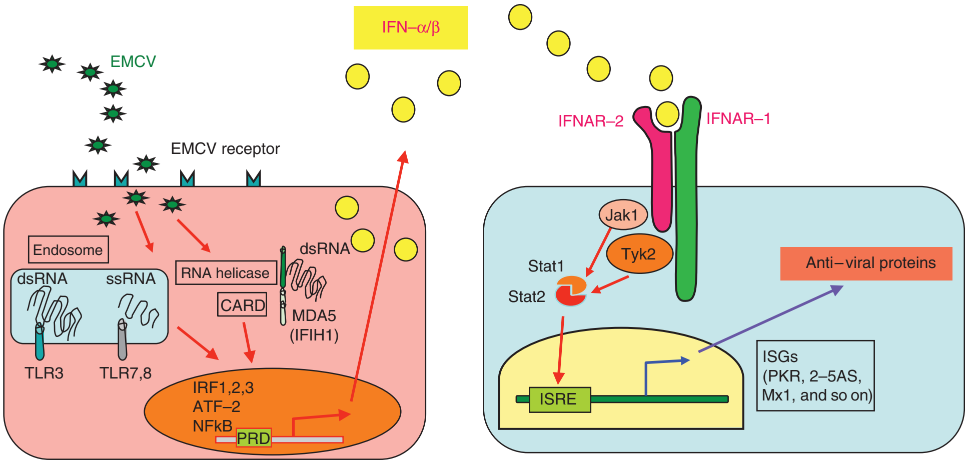

EMCV: encephalomyocarditis virus
IRF: interferon-releasing factor
PRD: positive regulatory domain
CARD: caspase recruitment domain
TLR: toll–like receptor
MDA: melanocyte differenciation antigen
(IFIH1: interferon induced with helicase C domain 1)
IFN: interferon

Jak1: Janus kinase 1
Tyk2: Tyrosine kinase 2
Stat: Signal transducers and activator or transcription
IFNAR: IFN-α/β receptor
ISRE: IFN-stimulated response element
ISGs: IFN-stimulated genes
PKR: RNA-dependent protein kinase
2–5AS: 2’ ,5’ -oligoadenylate synthetase
Mx1: IFN-induced GTP-binding protein Mx1

Figure 1 | Type 1 interferon (IFN-α/β) signalling pathway. Tyk2 and Jak1 are reciprocal IFN receptor-associated molecules, mediating the downstream signal to induce ISGs to resist against viral infection (modified from ref. 3, Fig. 5.3).

gene expression in pancreatic β-cells is crucial for providing resistance against EMC-D virus-induced diabetes.

Biological significance of IFN-α. EMC-D virus-infected *Tyk2* KO mice produced higher levels of IFN-α than *Tyk2* WT mice in both serum (Fig. 3a) and pancreas (Fig. 3b). Since IFN- or IFN-inducible poly I:C was reported to modify the outcome of experimental virus-induced diabetes[27,28], we further examined the biological significance of increased IFN-α. We initially examined the outcome of intraperitoneal adoptive transfer of 10^4 units IFN-α and found that transferred IFN-α appeared in the blood as early as 2 or 3 h after injection (Supplementary Fig. 2a). The IFN transfer did induce elevation of IFN-stimulated genes (ISGs) of the pancreatic islet-β cells (Supplementary Fig. 2b), suggesting that IFN-α transfer is effective to induce IFN-mediated responses in the islet-β cells. Treatment with IFN transfer alone did not affect the glucose levels in noninfected mice (Fig. 3c), suggesting that elevated IFN alone did not operate to damage pancreatic β-cells. Moreover, a high dose of IFN transfer did not alter the outcome of virus-induced diabetes in mice infected with EMC-D virus (Fig. 3d). These observations, taken together, suggest that enhanced production of IFN in *Tyk2* KO mice was induced by strong stimulation for the innate immune system to produce IFN, due to extensive proliferation of EMC-D virus (Fig. 2c), and also indicate that the increased IFN-α in *Tyk2* KO mice did not possess any biological significance to destroy β-cells or to resist against virus-induced diabetes.

***Tyk2* gene mutations in susceptible mice.** Since *Tyk2* gene KO mice were highly susceptible to EMC-D virus-induced diabetes as described above, we screened for mutations of the *Tyk2* gene in virus-induced diabetes-sensitive DBA/2J (DBA), SJL/J (SJL) and SWR/J (SWR) mice as well as resistant B6, C3H/HeJ and moderately susceptible A/J, and BALB/cJ mice. We found *Tyk2* gene mutations at the promoter region (− 678_ − 674 5A > 5T, − 713T > C, − 735 C > T, − 919 G > T, − 938_ − 930 del 9T, − 998 G > C, − 1010T > C, − 1015 C > T, − 1219A > G) from transcription start point at exon 1 and exons 9592A > G and 10642A > G only in virus-induced diabetes-sensitive SJL and SWR mice (Table 1). All virus-induced diabetes-resistant and moderately susceptible strains lack *Tyk2* gene mutations. Interestingly, highly virus-induced diabetes-susceptible DBA mice have the WT *Tyk2* gene (Table 1).

Significance of the mutated *Tyk2* gene in virus-induced diabetes. In order to determine the significance of the mutation of the *Tyk2* gene, we generated congenic mice that had a mutated (MT) *Tyk2* haplotype gene with resistant B6 genetic background. We also backcrossed B6 to SJL mice to obtain congenic mice with susceptible SJL background possessing the WT *Tyk2* gene. The congenic B6 mice, carrying the MT *Tyk2* gene, presented high sensitivity to EMC-D virus-induced diabetes (Fig. 4a) associated with extensive β-cell damage (Fig. 4b), while congenic SJL/J mice with the WT *Tyk2* gene showed resistance (Fig. 4c), thus providing evidence that the MT *Tyk2* gene is responsible for determining susceptibility to EMC-D virus-induced diabetes. Immunohistochemical study revealed that *Tyk2* WT mice had mild CD45-positive leukocyte infiltration with increased IFNα and upregulation of class I major histocompatibility complex (MHC) in 3 days after infection in the islets. Those inflammatory responses rapidly subsided in 5 days after infection, preserving almost intact islet β-cells (Supplementary Fig. 3a). In contrast, *Tyk2*-mutated mice developed significant CD45-positive leukocyte infiltration with increased IFNα and upregulation of class I MHC in 3 days after infection and persisted, leading to the extensive destruction of pancreatic β-cells as well as *Tyk2* KO mice (Supplementary Fig. 3a), suggesting that inflammatory response in the pancreatic islets after EMC-D virus infection may possibly enhance the islet cell damage in addition to virus-induced β-cell lysis. Production of IFN-β was observed only on 5 days after infection in the wide range of pancreatic cells in *Tyk2* KO mice (Supplementary Fig. 3a). Thus, pancreas tissue, but not infiltrating leukocytes, seemed to produce both IFN-α □□□ -β. Interestingly, *Tyk2* KO *MIP-Tyk2* Tg mice produced IFN-α in the islets □□□□□□□□□□ □□□ □□□□□ □□[illegible] □□□□, possibly because of high *Tyk2* gene expression in

© 2015 Macmillan Publishers Limited. All rights reserved.

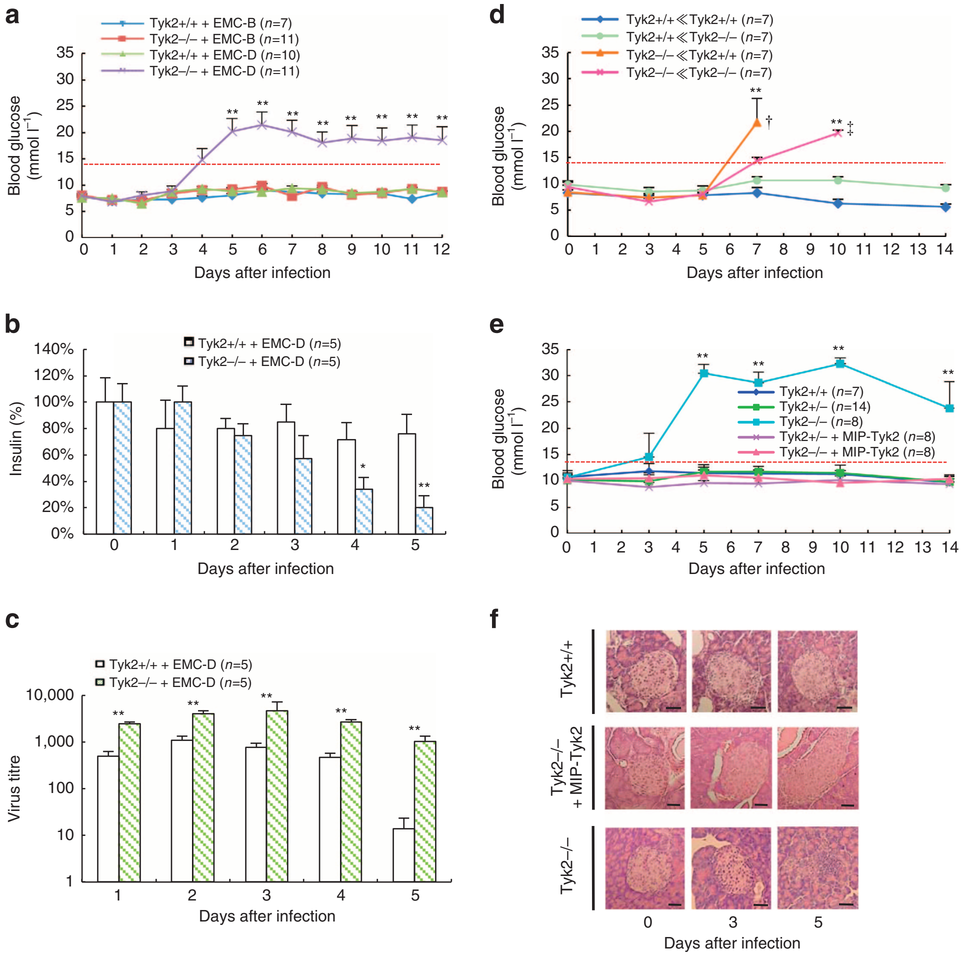

Figure 2 | Development of diabetes in EMC-D virus-infected *Tyk2* KO mice. (**a**) Blood glucose levels in WT (*Tyk2*+/+) and *Tyk2* KO (*Tyk2*−/−) mice with B6 genetic background, which was used throughout the study except indicated, infected with EMC-D or EMC-B virus. (**b**) Insulin content of the pancreas from *Tyk2*+/+ and *Tyk2*−/− mice infected with EMC-D virus. (**c**) Virus titres in the pancreas from *Tyk2*+/+ and *Tyk2*−/− mice infected with EMC-D virus. (**d**) Blood glucose levels of lethally irradiated *Tyk2*+/+ or *Tyk2*−/− mice reconstituted with *Tyk2*+/+ or *Tyk2*−/− spleen cells infected with EMC-D virus. The irradiated *Tyk2* WT recipient mice that were transferred spleen cells derived from either WT donor mice or *Tyk2* KO mice (*Tyk2*+/+ ≪ *Tyk2*+/+, or *Tyk2*+/+ ≪ *Tyk2*−/−), or the irradiated KO recipient mice received spleen cells derived from either WT donor mice or *Tyk2* KO mice (*Tyk2*−/− ≪ *Tyk2*+/+ or *Tyk2*−/− ≪ *Tyk2*−/−). †died by 10 days after infection, ‡died by 14 days after infection. (**e**) Blood glucose levels in *MIP-Tyk2* Tg *Tyk2*−/− (*Tyk2*−/− +*MIP-Tyk2*) mice infected with EMC-D virus. (**f**) Histopathology of pancreatic islets from *Tyk2*−/−, *Tyk2*+/+ and *MIP-Tyk2* Tg *Tyk2*−/− (*Tyk2*−/− +*MIP-Tyk2*) mice infected with EMC-D virus. Scale bar, 50 μm. In **a**,**d**,**e**, animals with blood glucose levels exceeding 14 mmol l^{-1} were diagnosed as diabetic. All data represent the mean ± s.e.m. Statistical significance (*$P<0.05$, **$P<0.01$) in **a**,**d**,**e** was determined by Kruskal–Wallis test with Bonferroni's *post hoc* analysis; in **b**,**c** by two-tailed Student's *t*-test in comparisons with *Tyk2*+/+, respectively.

pancreatic β-cells, and minimal leukocyte infiltrations on 5 days after infection, escaping from the virus-induced islet β-cell damage (Supplementary Fig. 3a). As shown in Supplementary Fig. 3b, transplanted *Tyk2*-deficient (KO) islets to the renal capsule of *Tyk2* WT mice did become infected with intraperitoneally injected EMC-D virus and were destroyed without insulin or glucagon (right), compared with noninfected transplanted islets with positive insulin and glucagon staining (left), suggesting that EMC-D virus infection reach systemically target organs and destroyed them when the target organ was susceptible to the viral infection.

Low *Tyk2* gene expression in mutated mice. Since we showed the significance of mutations of the *Tyk2* gene in the susceptibility to virus-induced diabetes, we next investigated whether mutations of the *Tyk2* gene affect expression level of the *Tyk2* gene. As shown in Fig. 5a, the expression of the *Tyk2* gene was almost absent in homozygous *Tyk2* gene-mutated (*Tyk2*$^{mt/mt}$) cells, as low as *Tyk2* KO (*Tyk2*$^{-/-}$) cells in all cell types studied, including mouse embryonic fibroblasts (MEFs), splenocytes and pancreatic β-cells. Restoration of β-cell-specific *Tyk2* gene expression was achieved by MIP-*Tyk2* transgene (Fig. 5a). In cells from heterozygous (*Tyk2*$^{+/-}$, *Tyk2*$^{+/mt}$) mice, *Tyk2* expression was moderately conserved (Fig. 5a). In addition, significantly reduced *Tyk2* promoter activity associated with *Tyk2* gene mutations at the promoter region was confirmed (Fig. 5b). These results suggest that the *Tyk2* MT gene observed in SJL/J mice was closely associated with a significant decrease in *Tyk2* gene expression level, possibly due to reduced promoter activity caused by multiple mutations at the *Tyk2* promoter region (Table 1). Since *Tyk2*-deficient mice produced high level of IFN (Fig. 2), we studied the IFN responses by IFN-inducible poly I:C treatment, coxsackie B4 virus, which belongs to a picorna virus group as well as EMC-D virus, infection, and herpes simplex virus (HSV), as a representative enveloped DNA virus, infection in various genotypes of mice, such as B6, SJL and *Tyk2*-mutated/B6. As a result, we found that treatment of poly I:C induced rapid IFN response

© 2015 Macmillan Publishers Limited. All rights reserved.

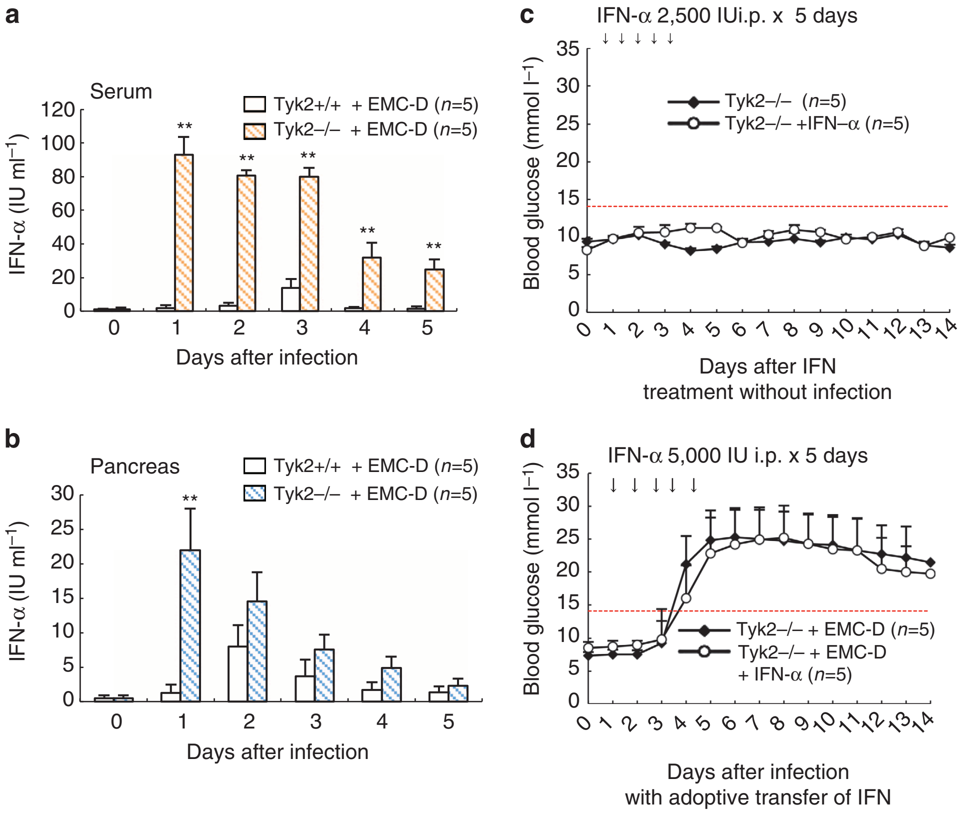

Figure 3 | Lack of biological importance of produced IFN-α in *Tyk2* KO mice. (**a**) IFN-α level in the serum. (**b**) IFN-α level in the pancreas. IFN-α produced in *Tyk2* KO (*Tyk2*−/−) and *Tyk2* WT (*Tyk2*+/+) mice on B6 background, infected with EMC-D virus were measured using ELISA. (**c**) Blood glucose levels after IFN-α treatment in *Tyk2*−/− and *Tyk2*+/+ mice without infection. Animals with blood glucose levels exceeding 14 mmol l^{-1} were diagnosed as diabetic. (**d**) Blood glucose levels following intraperitoneal injection of high dose IFN-α (5,000 IU) for five consecutive days, after EMC-D virus infection. All data represent the mean ± s.e.m. Statistical significance (*$P<0.05$ or **$P<0.01$) in **a**,**b** was determined by two-tailed Student's *t*-test; in **c**,**d** by Kruskal–Wallis test with Bonferroni's *post hoc* analysis.

Table 1 | Mutations of the *Tyk2* gene in virus-induced diabetes-susceptible mice.

Susceptibility of EMC-D virus-induced DM	Strain	Promoter region*	Exon 7	Exon 8
			9592A>G (H234R)	10642A>G (K355E)
Resistant	C57BL/6J	WT	WT	WT
	C3H/HeJ	WT	WT	WT
Moderately susceptible	BALB/cJ	WT	WT	WT
	A/J	WT	WT	WT
Highly susceptible	DBA/2J	WT	WT	WT
	SJL/J	MT	MT	MT
	SWR/J	MT	MT	MT

DM, diabetes mellitus; EMC-D, encephalomyocarditis virus D strain; MT, mutated type; WT, wild type.
*Mutations of *Tyk2* gene promoter region (MT) within −1,300 bp upstream from transcription start point were −678_−674 5A>5T, −713T>C, −735 C>T, −919 G>T, −938_−930 del 9T, −998 G>C, −1010T>C, −1015 C>T, −1219A>G.

(Supplementary Fig. 4a) in all strains of mice studied. Coxsackie B4 virus infection also enhanced IFN production in all strains (Supplementary Fig. 4b), while HSV infection scarcely induced IFN response (Supplementary Fig. 4c), suggesting that enveloped DNA virus may be a poor IFN inducer, at least in those strains of mice studied.

***Tyk2* gene mutation reduced antiviral effect**. In order to assess whether the deletion or mutation of the *Tyk2* gene leads to reduced expression of the *Tyk2* gene and reduced protective capability dependent on IFN against EMC-D virus-induced diabetes, we evaluated the expression levels of the *Tyk2* gene, *Jak1* gene and ISGs in pancreatic β-cells, splenocytes and embryonic fibroblasts (mEFs) with IFN treatment. We first studied the expression levels of *Tyk2* and *Jak1* genes in cells derived from *Tyk2* gene-deficient or -mutated mice. It was found that the expression levels of the *Tyk2* gene in *Tyk2*-mutated mice were as low as those of *Tyk2* KO mice before and after IFN treatment (Fig. 6a). We also studied the expression level of the *Jak1* gene, which is a signal transduction molecule associated with cytoplasmic domain of the IFN receptor, as well as Tyk2 (Fig. 1). Expression of the *Jak1* gene was not suppressed but well maintained in *Tyk2* KO or *Tyk2* MT mice (Fig. 6a), suggesting the alternative and/or supplemental role in *Tyk2*-deficient mice in mediating downstream signal transduction stimulated by IFN[24,29]. We further analysed the expression levels of ISGs induced by type I IFN. Before IFN-β stimulation, expression of all ISGs was almost absent in all cell types even from *Tyk2*$^{+/+}$ WT mice (Fig. 6b). Following IFN stimulation, expression of ISGs was significantly increased in cells from WT mice (Fig. 6b). Consistent with the expression levels of the *Tyk2* gene, the expression levels of ISGs were high in cells from *Tyk2*$^{+/+}$ WT mice, while they were markedly reduced in cells from *Tyk2* KO mice and *Tyk2* MT mice (Fig. 6b), with low but distinct expression of ISGs, possibly due to complementary *Jak*1-mediated partial signal transduction as described above (Fig. 6b). *MIP-Tyk2* transgene significantly

© 2015 Macmillan Publishers Limited. All rights reserved.

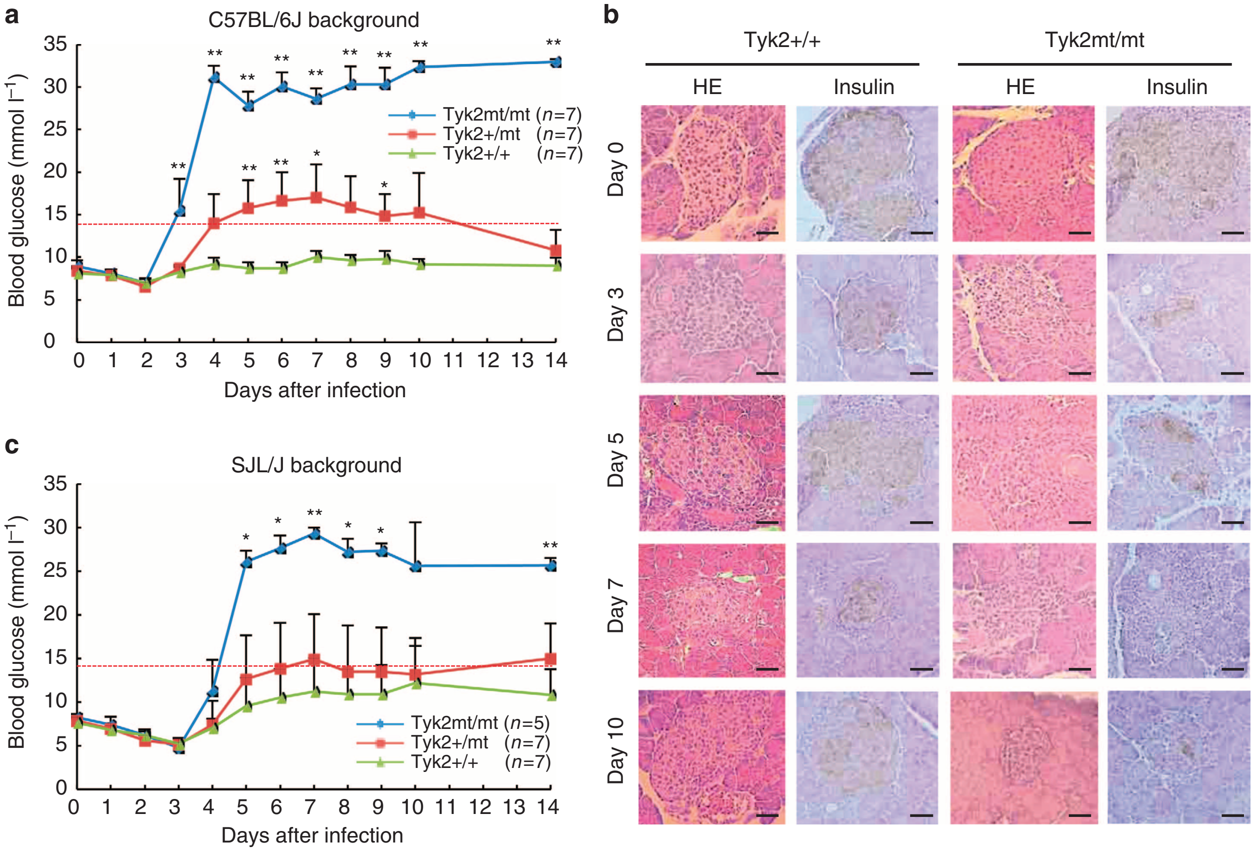

Figure 4 | Significance of the mutated *Tyk2* gene in congenic mice. (**a**) Blood glucose levels of B6 mice with *Tyk2*+/+, heterozygous mutated *Tyk2* (*Tyk2*+/mt) and homozygous mutated *Tyk2* gene (*Tyk2*mt/mt) infected with EMC-D virus. (**b**) Histopathology of pancreatic islets from *Tyk2*+/+ or *Tyk2*mt/mt mice with B6 background infected with EMC-D virus. Scale bar, 50 μm. (**c**) Blood glucose levels of in *Tyk2*+/+, *Tyk2*+/mt, *Tyk2*mt/mt gene carrying congenic mice, with SJL genetic background, infected with EMC-D virus. In **a**,**c**, animals with blood glucose levels exceeding 14 mmol l^{-1} were diagnosed as diabetic. All data represent the mean ± s.e.m. Statistical significance (*$P<0.05$, **$P<0.01$) in **a**,**c** was determined by Kruskal–Wallis test with Dunnett's *post hoc* analysis.

restored the decreased expression of *Tyk2* in ββ-cells, but not in splenocytes or mEF cells derived from *Tyk2*-deficient mice (Fig. 6b). These results suggest that the expression of *Tyk2* is involved in mediating the IFN signalling pathway and the induction of ISGs. Finally, we assessed the ability of IFN-β treatment to inhibit virus-induced cell damage *in vitro*. The inhibition of virus-induced cell death on stimulation with IFN-β was significantly reduced in mEF or islet cells derived from *Tyk2*-mutated mice, to levels as low as *Tyk2* KO mice (Fig. 6c). In addition, it should be noted that after treatment with a high concentration of IFN, *Tyk2* MT mEF cells could restore the reduced antiviral activity, while *Tyk2* MT β-cells sustained deteriorated the capability to resist against virus-induced cell death (Fig. 6c), indicating that β-cells were more sensitive than mEF cells in decreased IFN-dependent antiviral responses because of *Tyk2* gene mutation. The finding was consistent with the *in vivo* experiments above in EMC-D virus-infected *Tyk2* KO mice, which showed the ineffectiveness of an increased IFN level to rescue virus-induced β-cell damage, and failed to prevent the development of diabetes (Fig. 2a). Moreover, we could also show that β-cell-specific *Tyk2* gene expression using *Tyk2* MT mice crossed with *MIP-Tyk2* Tg mice (*MIP-Tyk2* Tg *Tyk2*$^{mt/mt}$) showed resistance to EMC-D virus-induced diabetes (Fig. 6d), similar to *MIP-Tyk2* Tg *Tyk2* KO mice, confirming the important role of intact *Tyk2* gene expression in pancreatic β-cells in the prevention of EMC virus-induced diabetes.

Discussion

In the present study, we could, for the first time to the best of our knowledge, identify the natural susceptibility gene as *Tyk2* to EMC virus-induced diabetes in mice. *Tyk2* gene mutation in diabetes-sensitive SJL and SWR mice is closely associated with significantly reduced *Tyk2* gene expression including that in pancreatic β-cells. It was suggested that the decreased *Tyk2* gene expression was due to decreased *Tyk2* promoter activity; however, other mechanisms may also be involved in the extensive suppression of *Tyk2* gene expression in these mutated mice. In addition, the importance of intact *Tyk2* gene expression in pancreatic β-cells in the resistance against EMC-D virus-induced diabetes was revealed by the *MIP-Tyk2* transgenic mice crossed with *Tyk2* KO or *Tyk2*-mutated mice. The observation is consistent with a previous report that showed the pancreatic β-cell-specific suppression of cytokine response including IFN leads to high sensitivity to coxsackie B4 virus infection[30], indicating that the appropriate cytokine response in pancreatic β-cells is most important to resist against virus-induced diabetes.

It should be noted that the *Tyk2*-deficient mice infected with EMC-D virus produced a high level of IFN but failed to rescue the mice from developing diabetes because high level of IFN could reverse the antiviral response in fibroblasts[24], but not pancreatic β-cells as shown by this study, suggesting the β-cell-specific unresponsiveness to a high level of IFN is critical in losing the capability to evade from virus-infected cell damage.

© 2015 Macmillan Publishers Limited. All rights reserved.

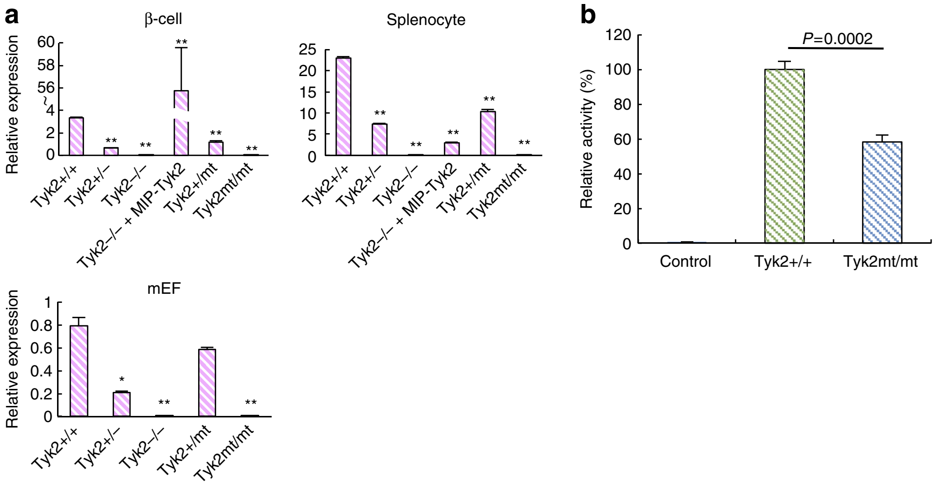

Figure 5 | Expression of Tyk2 protein and gene in *Tyk2* gene-mutated mice. (**a**) Relative expression levels of the *Tyk2* gene were measured by qRT–PCR (see Methods). Pancreatic β-cells, splenocytes and MEF cells were isolated from B6 mice with genotypes as indicated in the figure. (**b**) Luciferase assay using genes possessing *Tyk2*+/+ or *Tyk2*mt/mt promoter region. All data represent the mean ± s.e.m. Statistical significance (*$P<0.05$, **$P<0.01$) was determined by two-tailed Student's *t*-test in comparisons with *Tyk2*+/+, respectively.

Although this study could clarify the genetic cause of the elevated susceptibility to the highly diabetogenic strain of EMC-D virus, these mice are still resistant to non-diabetogeneic EMC-B virus (Fig. 2a)[13]. Clinical outcomes of infectious diseases are dependent on many factors, including pathogenicity of the pathogen and host resistance factors such as immunological resistance, age, sex and stochastic elements. Interestingly, the different diabetogenic potential of different strains of EMC viruses has been noted to be dependent on the genetic variation[31]. Although diabetogenic EMC-D virus and nondiabetogenic EMC-B virus could not be distinguished by either neutralization assay or competitive radioimmunoassay, only one amino-acid change, induced by only one nucleotide substitution, is critical to acquire diabetogenicity of the EMC virus[32]. These beautiful studies suggested that a single point mutation in a potential diabetogenic virus can easily acquire higher diabetogenicity in the natural state.

To prove the causality of the pathogen in inducing an infectious disease, historically and ideally, 'Koch's postulate' should be fulfilled[33]. The one organism–one disease paradigm is central to Koch's postulate, in which it is essentially required to show that an isolated pathogen can cause the disease in experimental animals[33]. However, this rule cannot be applied to all microbe-induced diseases. With respect to virus-induced diabetes, many viruses have been noted to be associated with the onset of diabetes[3,6–9,34]. However, distinguishing between whether the pathogen was a causal factor or was only a coincidental co-infection is difficult[3,10]. In addition, when cumulative environmental insults lead to the development of diabetes[35], virus infection may serve as one of the many risk factors that may contribute to diabetes development. Accordingly, only a few reports have proved the diabetogenicity of virus isolates from humans[36,37]. Both viruses were isolated by using SJL mice[36,37], and these studies appear to fulfill Koch's postulate. Since SJL mice carry the susceptible *Tyk2* gene mutation as shown by this study, the SJL mouse model may be an appropriate animal assay system to simulate human virus-induced diabetes susceptibility.

Among the susceptible strains of mice, DBA/2 mice, which are also highly susceptible to EMC-D virus-induced diabetes, lack *Tyk2* gene mutation, indicating that genes other than *Tyk2* may be responsible for increased susceptibility to virus-induced diabetes. Interestingly, it was reported that polymorphisms of the *IFIH1* gene, which is an intracellular pathogen recognition receptor for picornavirus including enteroviruses, operating as an inducer of IFN production[20], is associated with risk for type 1 diabetes[38]. Moreover, a rare *IFIH1* gene variant confer resistance to type 1 diabetes[39]. In addition, it was reported that MDA5 and TLR3 are both required to prevent diabetes in mice infected with EMC-D virus[23]. These observations taken together suggest that the *MDA5/IFIH1* gene may possibly be a common susceptibility gene to virus-induced diabetes in humans and mice. In order to develop mouse models with higher virus-induced diabetes susceptibility for use as an *in vivo* assay system to evaluate diabetogenicity of candidate viruses, the identification of genes associated with high susceptibility to virus-induced diabetes in DBA/2 mice or in humans including the *MDA5/IFIH1* gene needs further investigation. These studies may in future lead to the identification of the 'diabetogenic' virus.

Although many viruses are possibly associated with the induction of diabetes in humans, accumulating evidence has suggested that enterovirus group may be a major causal pathogen in virus-induced diabetes. Therefore, development of vaccines against diabetogenic enteroviruses will hopefully contribute to prevent a major portion of virus-induced diabetes in humans.

In conclusion, this manuscript reports the identification of the murine natural virus-induced diabetes susceptibility gene as *Tyk2*, and demonstrates that *Tyk2* gene expressed in pancreatic β-cells controls susceptibility to virus-induced diabetes.

Methods

Virus. EMC-B and -D virus was kindly provided by Dr A.L.Notkins, NIH, USA and J-W Yoon, University of Calgary, Canada. The viral titre was determined by plaque assay on mEF cells.

Mice. WT of C57BL/6J (B6), C3H/HeJ, BALB/c, A/J, DBA/2J, SJL/J and SWR/J J mice were purchased from Kyudo (Japan). *Tyk2* KO mice, having EMC-D virus-induced diabetes-resistant B6 genetic background, were generated by backcrossing 129/Sv-derived *Tyk2* KO mice[24] to B6 mice for 12 times. All strains of male mice aged 6–10 weeks were used throughout the study.

The *MIP-Tyk2* transgenic construct was assembled using an 8.5-kb fragment of the *MIP* that includes a region from −8.5 kb to −12 bp (relative to the transcriptional start site), the coding region of *Tyk2* (3.6 kb; Clontech, Palo Alto, CA) and a 2.1-kb fragment of the human *growth hormone* (*hGH*) gene cassette for high-level expression (ref. 26). The fragments of *MIP* and *hGH* cassette were from *MIP-EGFP-hGH* transgenic construct kindly provided by Dr M. Hara[27]. The 14.6-kb *MIP-Tyk2-hGH* fragment was isolated from the vector by digestion of

© 2015 Macmillan Publishers Limited. All rights reserved.

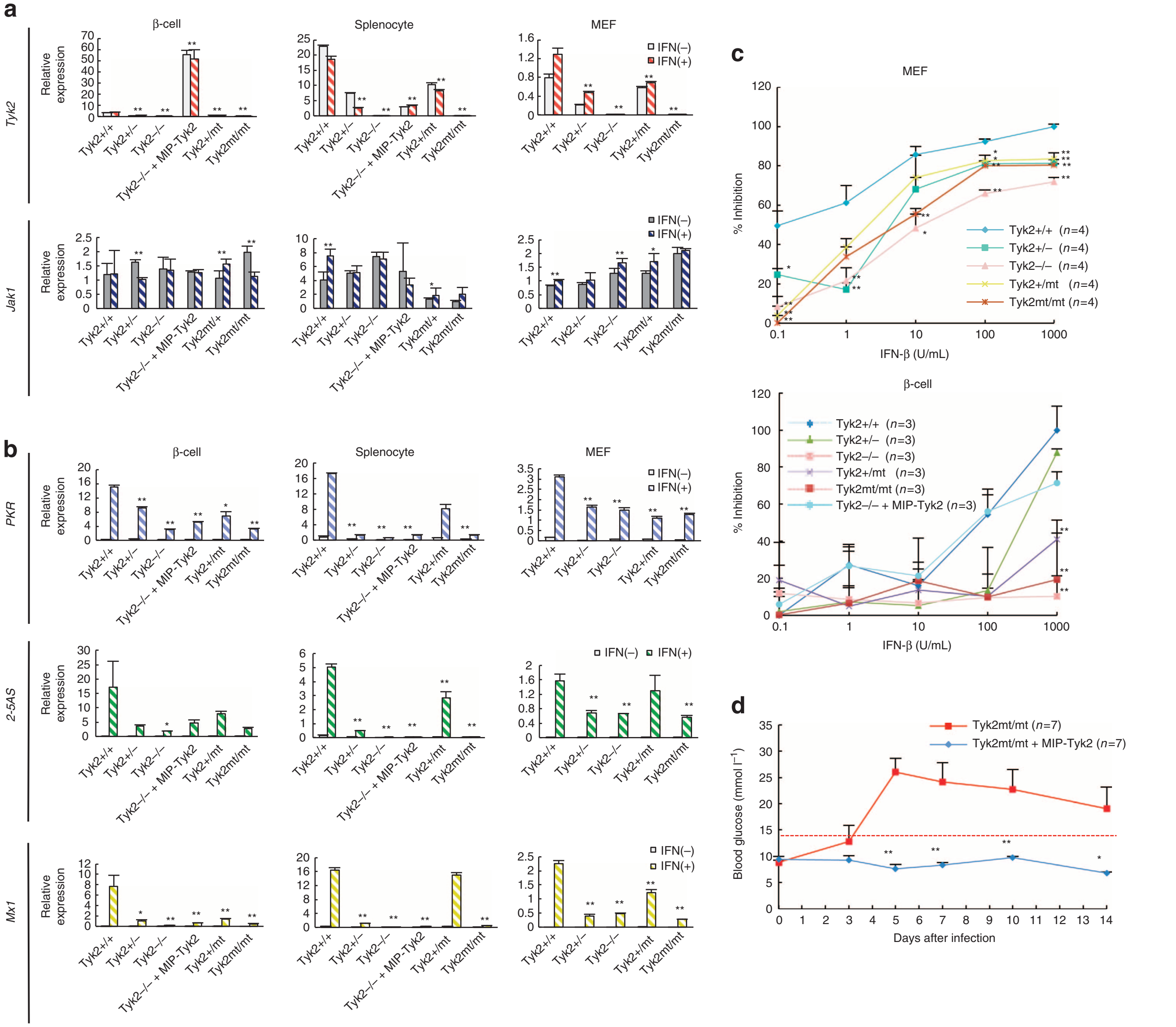

Figure 6 | Expression of IFN-*stimulated* genes and antiviral effect in IFN-treated cells. (**a**) Relative expression levels of the *Tyk2* and *Jak1* genes measured using qRT–PCR (See Methods) in IFN-β-stimulated pancreatic β-cells, splenocytes and MEF cells, isolated from various genotypes of mice with B6 background as indicated in the figure. (**b**) Relative expression levels of *ISGs (PKR, 2–5AS, Mx1)* measured by qRT–PCR in IFN-β-stimulated β-cells, splenocytes and MEF cells, isolated from B6 mice with genotypes as indicated in the figure. (**c**) IFN-dependent inhibitory activity against EMC-D virus-induced cell death in MEF or β-cells derived from mice with genotypes as indicated. (**d**) Blood glucose levels in *MIP-Tyk2* Tg *Tyk2*mt/mt (Tyk2mt/mt + MIP-Tyk2) mice infected with EMC-D virus. In **d**, animals with blood glucose levels exceeding 14 mmol l^{-1} were diagnosed as diabetic. In **a**,**b**, relative activity of each gene expression before and after IFN-β stimulation was shown. All data represent the mean ± s.e.m. Statistical significance (**P*<0.05, ***P*<0.01) in **a**,**b** were determined by two-tailed Student's *t*-test between IFN-β-stimulated each sample and that of Tyk2 + / +; in **c** by Kruskal–Wallis test with Dunnett's *post hoc* analysis; in **d** by Kruskal–Wallis test with one-way analysis of variance.

the plasmid construct with HindIII and PvuI and with agarose gel electrophoresis. The fragment was further purified using a QIAEXII Gel Extraction Kit (Qiagen, Valencia, CA). The purified transgene DNA was microinjected into the pronuclei of C57BL/6 mice at the Keio University, generating *MIP-Tyk2* Tg mice. In order to verify the specific expression of *Tyk2* in pancreatic β-cells, we compared the expression level of *Tyk2* in pancreatic β-cells and splenocytes. The mRNA level of *Tyk2* in islet cells was quantified by quantitative reverse transcription polymerase chain reaction (qRT–PCR).

In order to transfer *Tyk2* mutations in virus-induced diabetes-sensitive SJL mice on a resistant B6 genetic background, male SJL/J mice were serially backcrossed with female B6 mice for eight times. Similarly, B6 mice with the WT *Tyk2* gene were backcrossed to SJL mice for five times, generating SJL mice with the WT *Tyk2* gene. The heterozygous carriers of the *Tyk2* mutation, used as breeders at each backcross generation, were identified by sequencing the genomic DNA isolated from the tail. Congenic level of the *Tyk2* gene, specially around the mutated *Tyk2* gene, was studied using 384 single-nucleotide polymorphism (SNP) markers by Charles River Genetic Testing Services. Overall mean congenic rate was 97.6%. Congenic mice carrying mutated *Tyk2* gene, SNP marker Chro9-1 at 13259371 bp, near the *Tyk2* gene at 2110408 to 21131275 bp, and Chr9-3 distant from the *Tyk2* gene at 23780447 bp, were both from B6 mice. Therefore, at least less than 77851036 bp at near side and 2649292 bp at distant side were successfully replaced by those of B6 mice derived gene. Congenic level of 129/Sv-derived *Tyk2* gene KO mice with B6 background was 99.1%.

All of the procedures involving mice were approved by the Keio and Kyushu University Animal Care and Use Committees.

Infection of mice with EMC-D virus. Mice were intraperitoneally (i.p.), intravenously or orally infected with 0.2 ml PBS containing 1.0×10^3 plaque-forming unit per mouse of EMC-D virus. The mice were also intraperitoneally infected with coxsackie B4 virus and HSV that were clinical isolates derived from the affected patients.

Assessment of diabetes condition and measurement of IFN-α. Blood glucose was measured by the glucose oxidase method (glutest sensor; Sanwa, Japan), and

© 2015 Macmillan Publishers Limited. All rights reserved.

insulin was measured by an enzyme-linked immunosorbent assay (ELISA) commercial kit as a standard (Morinaga, Japan). Serum IFN-α was measured by an ELISA kit (Cosmo Bio, Japan). Mice with blood glucose levels greater than 14.0 mmol l^{-1} were diagnosed as diabetic.

Histological and immunohistochemical analyses. Pancreases were removed and rinsed with saline. The tissues were fixed with 10% formalin and embedded in paraffin. Tissue sections were cut at a thickness of 3.0 μm and stained with haematoxylin and eosin. For insulin stain, the paraffin-embedded sections were stained with anti-insulin antibody (Molecular Probes, Eugene, OR). The signals were detected by a Vectastain avidin–biotin–peroxidase complex system with a diaminobenzidine substrate kit (Vector Laboratories, Burlingame, CA). Immunohistochemical study was also carried out using frozen sections of the pancreas, followed by the fixation with 4% paraformaldehyde/PBS. To stain insulin, glucagon, IFN-α, IFN-β, CD45 and MHC class I. For insulin, guinea pig anti-pig insulin antibody (AbD Serotec, Kidlington, UK)and Alexa Fluor 488 goat anti-guinea pig IgG (Life Technologies, USA), for glucagon, rabbit glucagon antibody (Cell Signaling Technology, Danvers, USA) and Alexa Fluor 594 goat anti-rabbit IgG (Life Technologies), for IFN-α rabbit polyclonal antibody against mouse IFN alpha (PBL Assay Science, USA) and anti-rabbit IgG(H + L)F(ab′)2Fragment (Cell Signaling Technology), for IFN-β, rabbit anti-IFN-beta (AbD Serotec)and Alexa Fluor 594 goat anti-rabbit IgG (Life Technologies), for CD45, rat anti-CD45 antibody (Abcam, Cambridge, UK) and Alexa Fluor 488 goat anti-rat IgG(Life Technologies), for MHC class I, goat anti-MHC class antibody (Abcam) and Alexa Fluor 594 goat anti-rat IgG (Life Technologies) were used.

Glucose tolerance. The intraperitoneal glucose tolerance test (IPGTT) was performed in mice after fasting for over 12 h. Blood was obtained from the tip of the tail. After baseline blood glucose level measurements, animals received an intraperitoneal injection of 1.5 g glucose per kg body weight. Blood glucose level was measured at 0, 5, 10, 15, 20, 25, 30, 40, 50, 60, 90 and 120 min.

Measurement of virus replication. The virus concentrations of the pancreatic tissues from EMC-D virus-infected mice were determined with plaque assay using mEF cells, as described previously[15].

Treatment of mouse with IFN-α or poly I:C. We purchased recombinant mouse IFN-α (PBL Biochemical Laboratories, Piscataway, NJ) and poly I:C (Sigma-Aldrich, Saint Louis, MI). We dissolved poly I:C in PBS in various concentrations and injected mice i.p. We used IFN alpha with Dulbecco's modified Eagle medium (DMEM) containing 5% fetal bovine serum and 1% penicillin/streptomycin (PcSM; Gibco, Carlsbad, CA). When the sample was determined to contain 10 IU of mouse IFN-α measured by the ELISA method, it was equivalent to 52.4 pg of IFN-α.

Anti-EMC-D virus activity. Antiviral activity was evaluated by CellTiter96 Aqueous (Promega, Madison, WI). mEF cells were cultured in a 96-well culture plate, 4×10^5 cells per well, and after 12 h serial dilutions of IFN-β (Sigma-Aldrich) were added. After 12 h, the cells were washed with PBS and cultured with EMC-D virus media. After an hour, virus media were removed and culture media added. Twenty-four hours later, 20 μl per well One Solution Reagent was added to the media and cell viability was determined at 490 nm after a 3-h incubation.

Splenic chimera mice. On day 0, recipient mice were lethally irradiated with 8.0 Gy whole-body irradiation (MBR-1520R-3, Hitachi, Japan) and then the animals were intravenously injected with 2×10^7 splenocytes on the same day. Forteen days later, mice were infected with EMC-D virus.

Transplantation of islets. Islets were isolated from *Tyk2* KO or WT mice and transplanted into the renal capsule of B6 mice.

Genetic variant analysis of the *Tyk2* gene at promoter region and exons. The variations in *Tyk2* promoter, Exon7 and Exon8 region, were analysed by cycle sequencing (BigDye Terminator v1.1 Cycle Sequencing Kit, ABI 3130 × l DNA Sequencer, Applied Biosystems, Carlsbad, CA) according to the manufacturer's protocol. We used the following primer sets to amplify the intended region of genomic DNA: for promoter region, forward 5′-CCAGGGCTTTCTGGTTT GTA-3′ and reverse 5′-CTATCTGGCCCAGGCAATAA-3′; for Exon7, forward 5′-CAGCTGCTCATCATGTCACC-3′ and reverse 5′-AAGCCAGGGTAGCTCAA GTG-3′; for Exon8, forward 5′-TGGGCTCAGTTTCCTTCTGT-3′ and reverse 5′-GAACAGAGGCACAGCAACTG-3′. We used following primers to sequence for 238 to − 590 (relative to the transcriptional start site) of the promoter region, 5′-CACTCACCGTTTCCGGAGTA-3′; for − 560 to − 713, 5′-TCAATTCCCAG CAACCACAT-3′; for − 793 to − 957, 5′-GTGGTTGCACATGCCTGTAA-3′; for − 668 to − 876, 5′-CTGTAGACCAGGCTGGCTTC-3′; for − 936 to − 1288, 5′-CCAGGGCTTTCTGGTTTGTA-3′; for − 941 to − 1346, 5′-CAGCTGAAG GGACAGTCTCA-3′; for Exon7, 5′-AAGCCAGGGTAGCTCAAGTG-3′; for Exon8, 5′-GAACAGAGGCACAGCAACTG-3′.

Cell culture and stimulation. Islet cells[40] and splenocytes were isolated from *Tyk2*$^{+/+}$, *Tyk2*$^{+/-}$, *Tyk2*$^{-/-}$, *Tyk2*$^{mt/+}$, *Tyk2*$^{mt/mt}$, *MIP-Tyk2* Tg mice. The mEF cells were obtained by homogenizing *Tyk2*$^{+/+}$, *Tyk2*$^{+/-}$, *Tyk2*$^{-/-}$, *Tyk2*$^{mt/+}$, *Tyk2*$^{mt/mt}$ mice embryos. All mEF cells were used before the fifth passage. Islet cells, splenocytes and mEF cells were maintained in DMEM (Gibco), RPMI medium 1640 (Gibco) and DMEM + GlutaMAX-1 (Gibco), supplemented with 10% fetal bovine serum (PAA) and 1% PcSM (Gibco), respectively. Islet cells, splenocytes and mEF cells were stimulated with IFN-β (500 U ml^{-1}; Sigma-Aldrich) for 12 h. Islet cells, splenocytes and mEF cells from *Tyk2*$^{+/+}$, *Tyk2*$^{+/-}$, Tyk2$^{-/-}$, Tyk2$^{mt/+}$, *Tyk2*$^{mt/mt}$ mice, and islet cells and splenocytes from *MIP-Tyk2* Tg mice were stimulated with IFN-β (500 U ml^{-1}) for 12 h. After 12 h, the cells were collected, and total cellular RNA was isolated to measure the expression of ISGs (*Pkr, 2–5AS, Mx1*) induced by IFN-β stimulation.

RNA extraction, cDNA reverse transcription and qRT–PCR analysis. RNA was extracted using ISOGEN (Nippon Gene, Japan) according to the manufacturer's instructions. The total RNA was reverse-transcribed into cDNA by using a High Capacity cDNA Reverse Transcription Kit (Applied Biosystems). The qRT–PCR was carried out by using the ABI 7500 Real-Time PCR system with Power SYBR green Master Mix (Applied Biosystems). The threshold cycle (C_t) value was normalized to that of *Glyceraldehyde-3-phosphate dehydrogenase* (*Gapdh*). The qRT–PCR was performed by using the following primer pairs: for *Tyk2*, forward 5′-AGCCATCTTGGAAGACAGCAA-3′ and reverse 5′-GACTTTGTGTGCGAT GTGGAT-3′; for Jak1, forward 5′-AGCATGGTATCTCTCCTCTCTG-3′ and reverse 5′-GATTCGGTTCGGAGCGTACC-3′; for *Pkr*, forward 5′-CCTCAGAGA ACGTGTTTACG-3′ and reverse 5′-TCAATTCTGTGTTTCGCTTT-3′; for *2–5AS*, forward 5′-CCCCATCTGCATCAGGAGGTGGAG-3′ and reverse 5′-AAGTCAT AATACTTTGTCCAGTAG-3′; for *Mx1*, forward 5′-CTGAGATGACCCAGCAC CTGAA-3′ and reverse 5′-CTCCAGGAACCAGCTGCACTTAC-3′; for *Gapdh*, forward 5′-AGGTCGGTGTGAACGGATTTG-3′ and reverse 5′-TGTAGACCAT GTAGTTGAGGTCA-3′.

Promoter activity analysis. The PCR-amplified *Tyk2* promoter fragment, either WT or mutant type sequences, was cloned into a pGL4.17[luc2/Neo] vector (Promega). WT and mutant type *Tyk2* promoter fragments were prepared from C57BL/6J and SJL/J mice, respectively, by PCR amplification using forward primer 5′-CCCGGTACCCCTTGGGACCTTTGGAGAAT-3′ and reverse primer 5′-CCC GCTAGCCACTCACCGTTTCCGGAGTA-3′, and then Kpn1 and Nhe1 were cut, leading to a 4,167-bp product. Luciferase assay was conducted by transfection of vectors to 293T cells with 24-well culture plates. Luciferase activity was measured after 24-h transfection in 293T cells using a dual luciferase assay kit (Promega). The experiments were repeated four times.

Statistical analysis. Statistical analyses were performed with the JMP9.0.2 software (SAS, Cary, NC). Differences between the means were assessed by two-tailed Student's *t*-test. Other differences among multiple means were assessed using several appropriate methods for each experiment, as by Kruskal–Wallis test with Bonferroni's *post hoc* analysis, by Kruskal–Wallis test with Dunnett's *post hoc* analysis or by one-way analysis of variance with Dunnett's *post hoc* analysis. Error bars represent the s.e.m. Null hypotheses were rejected at the 0.05 level.

References

1. Scully, T. Diabetes in numbers. *Nature* **485,** S2–S3 (2012).
2. Jonietz, E. Cause and effect. *Nature* **485,** S10–S11 (2012).
3. Taylor, K. W. in *Diabetes and Viruses.* (eds. Taylor, K. W., Hyöty, H., Toniolo, A. & Zuckerman, A.) (Springer Science + Business Media 3–6, 2013).
4. Coppieters, K. T., Boettler, T. & von Herrath, M. Virus infections in type 1 diabetes. *Cold Spring Harb. Perspect. Med.* **2,** 1–14 (2012).
5. Jenson, B. A., Rosenberg, H. S. & Notkins, A. L. Pancreatic islet-cell damage in children with fatal viral infections. *Lancet* **II,** 354–358 (1980).
6. Clements, G. B., Galbraith, D. N. & Taylor, K. W. Coxsackie B virus infection and onset of childhood diabetes. *Lancet* **346,** 221–223 (1995).
7. Dahlquist, G. G., Ivarsson, S., Lindberg, B. & Forsgren, M. Maternal enteroviral infection during pregnancy as a risk factor for childhood IDDM. A population-based case-control study. *Diabetes* **44,** 408–413 (1995).
8. Tanaka, S. *et al.* Enterovirus infection, CXC chemokine ligand 10 (CXCL10), and CXCR3 circuit: a mechanism of accelerated beta-cell failure in fulminant type 1 diabetes. *Diabetes* **58,** 2285–2291 (2009).
9. Tauriainen, S., Oikarinen, S., Oikarinen, M. & Hyöty, H. Enteroviruses in the pathogenesis of type 1 diabetes. *Semin. Immunopathol.* **33,** 45–55 (2011).
10. Jun, H. S. & Yoon, J. W. The role of viruses in type I diabetes: two distinct cellular and molecular pathogenic mechanisms of virus-induced diabetes in animals. *Diabetologia* **44,** 271–285 (2001).
11. Craighead, J. E. & McLane, M. F. Diabetes mellitus: induction in mice by encephalomyocarditis virus. *Science* **162,** 913–914 (1968).
12. Nagafuchi, S., Kurisaki, H. & Katsuta, H. in *Diabetes and Viruses.* (eds Taylor, K. W., Hyöty, H., Toniolo, A. & Zuckerman, A.) (Springer Science + Business Media 37–48, 2013).

© 2015 Macmillan Publishers Limited. All rights reserved.

13. Yoon, J. W., McClintock, P. R., Onodera, T. & Notkins, A. L. Virus-induced diabetes mellitus. XVIII. Inhibition by a nondiabetogenic variant of encephalomyocarditis virus. *J. Exp. Med.* **152,** 878–892 (1980).
14. Kang, Y. & Yoon, J. W. A genetically determined host factor controlling susceptibility to encephalomyocarditis virus-induced diabetes in mice. *J. Gen. Virol.* **74,** 1207–1213 (1993).
15. Kounoue, E. *et al.* The significance of T cells, B cells, antibodies and macrophages against encephalomyocarditis (EMC)-D virus-induced diabetes in mice. *Arch. Virol.* **153,** 1223–1231 (2008).
16. Ross, M. E., Onodera, T., Brown, K. S. & Notkins, A. L. Virus-induced diabetes mellitus. IV. Genetic and environmental factors influencing the development of diabetes after infection with the M variant of encephalomyocarditis virus. *Diabetes* **25,** 190–197 (1976).
17. Yoon, J. W., McClintock, P. R., Bachurski, C. J., Longstreth, J. D. & Notkins, A. L. Virus-induced diabetes mellitus. No evidence for immune mechanisms in the destruction of beta-cells by the D-variant of encephalomyocarditis virus. *Diabetes* **34,** 922–925 (1985).
18. Onodera, T., Yoon, J. W., Brown, K. S. & Notkina, A. L. Evidence for a single locus controlling susceptibility to virus-induced diabetes mellitus. *Nature* **274,** 693–696 (1978).
19. Boucher, D. W. & Notkins, A. L. Virus-induced diabetes mellitus I. Hyperglycemia and hypoinsulinemia in mice infected with encehphalomyocarditis virus. *J. Exp. Med.* **137,** 1226–1239 (1973).
20. Takeuchi, O. & Akira, S. Innate immunity to virus infection. *Immunol. Rev.* **227,** 75–86 (2009).
21. Ishikawa, H., Ma, Z. & Barber, G. N. STING regulates intracellular DNA-mediated, type I interferon-dependent innate immunity. *Nature* **461,** 788–792 (2009).
22. Sun, L., Wu, J., Du, F., Chen, X. & Chen, Z. J. Cyclic GMP-AMP synthase is a cytosolic DNA sensor that activates the type I interferon pathway. *Science* **339,** 786–791 (2013).
23. McCartney, S. A. *et al.* RNA sensor-induced type I IFN prevents diabetes caused by a beta cell-tropic virus in mice. *J. Clin. Invest.* **121,** 1497–1507 (2011).
24. Shimoda, K. *et al.* Tyk2 plays a restricted role in IFN alpha signaling, although it is required for IL-12-mediated T cell function. *Immunity* **13,** 561–571 (2000).
25. Mein, C. A. *et al.* A search for type 1 diabetes susceptibility genes in families from the United Kingdom. *Nat. Genet.* **19,** 297–300 (1998).
26. Hara, M. *et al.* Transgenic mice with green fluorescent protein-labeled pancreatic beta-cells. *Am. J. Physiol. Endocrinol. Metab.* **284,** E177–E183 (2003).
27. Gould, C. L., McMannama, K. G., Bigley, N. J. & Giron, D. J. Exacerbation of the pathogenesis of the diabetogenic variant of encephalomyocarditis virus in mice by interferon. *J. Interferon Res.* **5,** 33–37 (1985).
28. Ewel, C. H., Sobel, D. O., Zeligs, B. J. & Bellanti, J. A. Poly I:C accelerates development of diabetes mellitus in diabetes-prone BB rat. *Diabetes* **41,** 1016–1021 (1992).
29. Karaghiosoff, M. *et al.* Partial impairment of cytokine responses in Tyk2-dificient mice. *Immunity* **13,** 549–560 (2000).
30. Flodström, M. *et al.* Target cell defense prevents the development of diabetes after viral infection. *Nat. Immunol.* **3,** 373–382 (2002).
31. Bae, Y. S., Eun, H. M. & Yoon, J. W. Molecular identification of diabetogenic viral gene. *Diabetes* **38,** 316–320 (1989).
32. Jun, H.-S., Kang, Y., Notkins, A. L. & Yoon, J. W. Gain or loss of diabetogenicity resulting frm a single point mutation in recombinant encephalomyocarditis virus. *J. Virol.* **71,** 9782–9785 (1997).
33. Tabrah, F. L. Koch's postulates, carnivorous cows, and tuberculosis today. *Hawaii Med. J.* **70,** 144–148 (2011).
34. Dotta, F. *et al.* Coxsackie B4 virus infection of beta cells and natural killer cell insulitis in recent-onset type 1 diabetic patients. *Proc. Natl Acad. Sci. USA* **104,** 5115–5120 (2007).
35. Toniolo, A., Onodera, T., Yoon, J. W. & Notkins, A. L. Induction of diabetes by cumulative environmental insults from viruses and chemicals. *Nature* **288,** 383–385 (1980).
36. Yoon, J. W., Austin, M., Onodera, T. & Notkins, A. L. Isolation of a virus from the pancreas of a child with diabetic ketoacidosis. *N. Engl. J. Med.* **300,** 1173–1179 (1979).
37. Champsaur, H. *et al.* Diabetes and Coxsackie virus B5 infection. *Lancet* **1,** 251 (1980).
38. Smyth, D. J. *et al.* A genome-wide association study of nonsynonymous SNPs identifies a type 1 diabetes locus in the interferon-induced helicase (IFIH1) region. *Nat. Genet.* **38,** 617–619 (2006).
39. Nejentsev, S., Walker, N., Riches, D., Egholm, M. & Todd, J. A. Rare variants of IFIH1, a gene implicated in antiviral responses, protect against type 1 diabetes. *Science* **324,** 387–389 (2009).
40. Katsuta, H. *et al.* Single pnacreatic beta cells co-express multiple islet hormone genes in mice. *Diabetogogia* **53,** 128–138 (2010).

Acknowledgements

We thank Itsuka Matsumoto, Ai Umei, Miki Kawano, Makoto Matsuo, Hiroko Hamada, Yumi Kamada, Yasuko Kajiki, Kanade Tokuzumim, Yukari Shimoda, Takamitsu Kasagi, Airsa Moroishi and Drs Yuji Ueki, Kazunobu Amako, Kaoru Aida and Tetsuro Kobayashi for their help in conducting this research. We also thank Drs Angela Koh and Chiri Nagatsuka for their help in preparing the manuscript. We appreciate the technical support from the Research Support Center, Graduate School of Medical Sciences, Kyushu University. This work was supported by grants (19209037 and 21659230) from the Ministry of Education, Culture, Sports, Science and Technology and a grant from the Ministry of Health, Labor, and Welfare of Japan.

Author contributions

K.I., T.Y., H.M., S.F. and S.N. prepared viruses including EMC-D virus, EMC-B virus, Coxsackie B4 virus and HSV. K.I., H.Y., Y.Y., K.A., Y.Y. and K.S. are involved in the studies regarding metabolic and immunologic conditions of virus-induced diabetes in *Tyk2* KO mice, generating congenic *Tyk2* KO mice. Y.Kai. and Y.Kurafuji. conducted the radiation transfer experiments. K.I., A.I. and D.M. did histopathologic and immunohistochemical studies of pancreas tissue. T.K., M.H. and H.K. generated *MIP-Tyk2* Tg mouse and studied the significance of beta-cell-specific restoration of the *Tyk2* gene in *Tyk2* KO and mutated mice. K.M. and S.N. performed the *Tyk2*-mutated congenic mouse research involving susceptibility to virus-induced diabetes, promoter function of mutated Tyk2 gene and IFN-induced antiviral responses *in vivo* and *in vitro*. M.T., K.H., H.K. and H.I. assessed the quantitative gene expressions including *Tyk2, Jak1 and interferon-stimulated genes*. K.I., K.M., K.A., H.K. and S.N. designed the experiments and wrote the manuscript. All authors read, gave comments to and agreed with the manuscript and Supplementary Information.

Additional information

Supplementary Information accompanies this paper at http://www.nature.com/naturecommunications

Competing financial interests: The authors declare no competing financial interests.

Reprints and permission information is available online at http://npg.nature.com/reprintsandpermissions/

How to cite this article: Izumi, K. *et al.* Reduced *Tyk2* gene expression in β-cells due to natural mutation determines susceptibility to virus-induced diabetes. *Nat. Commun.* 6:6748 doi: 10.1038/ncomms7748 (2015).

This work is licensed under a Creative Commons Attribution 4.0 International License. The images or other third party material in this article are included in the article's Creative Commons license, unless indicated otherwise in the credit line; if the material is not included under the Creative Commons license, users will need to obtain permission from the license holder to reproduce the material. To view a copy of this license, visit http://creativecommons.org/licenses/by/4.0/

© 2015 Macmillan Publishers Limited. All rights reserved.
